# NLRP3 Inflammasome’s Activation in Acute and Chronic Brain Diseases—An Update on Pathogenetic Mechanisms and Therapeutic Perspectives with Respect to Other Inflammasomes

**DOI:** 10.3390/biomedicines11040999

**Published:** 2023-03-23

**Authors:** Anna Chiarini, Li Gui, Chiara Viviani, Ubaldo Armato, Ilaria Dal Prà

**Affiliations:** 1 Human Histology & Embryology Section, Department of Surgery, Dentistry, Pediatrics, and Gynecology, University of Verona, 37134 Verona, Italy; chiaraviviani.3@gmail.com (C.V.); uarmato@gmail.com (U.A.); 2 Department of Neurology, Southwest Hospital, Chongqing 400038, China; 2079244086@email.szu.edu.cn

**Keywords:** neuroinflammation, inflammasomes, NLRP3, inhibitors, brain, neurodegenerative diseases, virus encephalitis, innate immunity

## Abstract

Increasingly prevalent acute and chronic human brain diseases are scourges for the elderly. Besides the lack of therapies, these ailments share a neuroinflammation that is triggered/sustained by different innate immunity-related protein oligomers called inflammasomes. Relevant neuroinflammation players such as microglia/monocytes typically exhibit a strong NLRP3 inflammasome activation. Hence the idea that NLRP3 suppression might solve neurodegenerative ailments. Here we review the recent Literature about this topic. First, we update conditions and mechanisms, including RNAs, extracellular vesicles/exosomes, endogenous compounds, and ethnic/pharmacological agents/extracts regulating NLRP3 function. Second, we pinpoint NLRP3-activating mechanisms and known NLRP3 inhibition effects in acute (ischemia, stroke, hemorrhage), chronic (Alzheimer’s disease, Parkinson’s disease, Huntington’s disease, MS, ALS), and virus-induced (Zika, SARS-CoV-2, and others) human brain diseases. The available data show that (i) disease-specific divergent mechanisms activate the (mainly animal) brains NLRP3; (ii) no evidence proves that NLRP3 inhibition modifies human brain diseases (yet ad hoc trials are ongoing); and (iii) no findings exclude that concurrently activated other-than-NLRP3 inflammasomes might functionally replace the inhibited NLRP3. Finally, we highlight that among the causes of the persistent lack of therapies are the species difference problem in disease models and a preference for symptomatic over etiologic therapeutic approaches. Therefore, we posit that human neural cell-based disease models could drive etiological, pathogenetic, and therapeutic advances, including NLRP3’s and other inflammasomes’ regulation, while minimizing failure risks in candidate drug trials.

## 1. Introduction

### 1.1. An Overall Picture

Acute and chronic human brain diseases have been attracting the increased attention of scientists and the public. This has been due to the concurrence of several factors, i.e., brain illnesses’ mounting prevalence, the persistent lack of effective therapies, increasingly huge healthcare and economic costs, hardships in assisting such patients particularly at home, marked psychopathological impacts on patients and relatives, a greater sensitivity to improper lifestyle consequences, and a common aspiration to long-lasting and healthy aging. To this must be added the growing concern about the serious risk that severe acute brain injuries surreptitiously evolve into chronic neuropathologies such as Alzheimer’s disease (AD), Parkinson’s disease (PD), and amyotrophic lateral sclerosis (ALS). Worldwide yearly estimates of acute brain injuries total about 42 million cases, while symptomatic AD by itself affects more than 50 million people. It is predicted that such figures will double or treble in twenty/thirty years unless effective therapies become available [1,2]. Yet, the latter quite understandable wish is hampered by ongoing controversies due to the still unclarified underlying pathogenetic mechanisms. A common feature in all brain diseases is ongoing neuroinflammation. From this observation, the hypothesis has been put forward that this inflammation is a main causative factor, whose mitigation or suppression would slow or stop the progression and/or improve the outcome [3,4]. 

“Inflammation” is a physiological defensive reaction of living tissues to harm, aiming at ridding the causative factor(s), disposing of cell debris, and restoring tissue integrity and homeostasis in the short term. In his treatise “*De Medicina*”, Roman physician Aulus Cornelius Celsius (~14–37 AD; [5]) first described acute inflammation’s five cardinal symptoms, i.e., “*rubor*” (Lat. reddening) and “*calor*” (Lat. heat), due to local increases in blood flow; “*tumor*” (Lat. swelling) caused by edema and leukocyte infiltration due to altered vessel permeability; “*dolor*” (Lat. pain), elicited by local acidosis overstimulating the nerves; and “*laesa functio*” (Lat. impaired function”), the injury’s downstream upshot. Conversely, a persisting (chronic) inflammation is a pathological condition whose upshot can be severe. 

Obviously, neuroinflammation has specific features, particularly in the various neurodegenerative diseases. In the latter, its onset can be early (familial cases) or surreptitious (sporadic cases). Its course is often quite slow, so that it can progress undetected for decades. However, while unnoticed, chronic neuroinflammation spreads from the site of origin (e.g., frontotemporal cerebral cortex, hippocampus, locus coeruleus, spinal cord) to other regions and in so doing progressively destroys the brain’s neuronal functional reserve. When the reserve is depleted, the gray matter of the cerebral cortex, basal ganglia, thalamus, brain stem, cerebellum, spinal cord, and the white matter connectome (axons) are remarkably thinned. At this stage, the diseases become symptomatic. Progressive decreases in abilities, such as memory, cognition, emotions, psychic, and motor activities, render the patients unable to cope. Eventually, the neuropathology inexorably and more rapidly moves toward the *obitus* [6,7]. The etiologic factors also trigger various collateral cellular processes, such as the overproduction of hydroxyl radicals, superoxide anions (reactive oxygen species or ROS), nitric oxide (NO), peroxynitrite, ionic dyshomeostasis, mitochondrial, lysosomal, and autophagy disfunctions, and overproduction and accumulation of toxic protein species, which sustain the neuroinflammation. Other events concur, such as leukocyte infiltration and alterations in blood–brain barrier (BBB) function. Altogether, such *noxae* drive positive feedback loops, aggravating the neuropathology [8,9,10,11,12,13]. 

Since Celsius’s time, and particularly in the last century, a huge amount of knowledge has been accumulating about the crucial relation between inflammation’s drivers and immunity. Nowadays, we know that the innate immune system secures the first protection against harmful factors or “molecular patterns”. The endogenous damage-associated molecular patterns (DAMPs) and homeostasis-altering molecular patterns (HAMPs) are sterile compounds (e.g., ATP, mitochondrial DNA), dysfunctional metabolism products, and cell debris. The exogenous pathogen-associated molecular patterns (PAMPs) are infectious (bacteria, fungi, viruses, prions) or toxic agents (chemicals, organic molecules). DAMPs/HAMPs/PAMPs form complexes with multiligand cellular “pattern recognition receptors” (PRRs). In turn, such complexes nucleate the assembly of multicomponent protein platforms, the “inflammasomes” [4,14], the activated signaling of which drives the tissue inflammation at the injury’s site.

#### NLRs Assembly and Signaling Activation

The PRRs’ group names are based upon shared structural domains. The most noted PRRs comprise the NLRs (NOD-like nucleotide-binding domain and leucine-rich-repeat (LRR) family of receptors); ALRs (absent in melanoma 2 receptors); and MEFV gene-encoded PYRIN receptors [15]. Currently, activated NLRs are the most intensely studied PRRs. In humans, NLRs having a PYRIN N-terminal homology domain (PYD) include 14 members, namely, NLRP1–NLR14. Physiologically, NLRs (excepting brain NLRs) keep an auto-inhibited conformation that winds up when they detect DAMPs/PAMPs/HAMPs. This drives the assembly and signaling activation of inflammasomes. NLRs’ N-terminal PYDs bind and nucleate the oligomerizing adaptor protein ASC (apoptosis-associated speck-like protein endowed with a caspase recruitment domain or CARD) [15,16]. Notably, the ASC gene encodes both a CARD and a PYD domain. Therefore, via CARD•CARD or PYD•PYD homotypic interactions, ASC proteins make complexes with the PYD or CARD domains of NLRs. PYDs and CARDs are conserved domains of 80–90 amino acids arranged in six anti-parallel α-helices forming an inner hydrophobic core with charged residues at the surface. Via CARD•CARD interactions, ASCs of canonical inflammasomes nucleate the inactive zymogens of caspase-1, a cysteine-type peptidase, causing their polymerization and proximity-mediated auto-catalytic self-cleavage, resulting in active caspase-1 duplets [16,17]. The latter produce mature interleukin (IL)-1β and IL-18 from their respective precursors and N-termini fragments of the gasdermin D protein (human, GSDMD; rodent, GsdmD), in addition to cleaving other proteins that share the YVHD/FESD consensus sequence [18]. Next, the GSDMD/GsdmD’s N-fragments oligomerize, forming transmembrane pores that extracellularly release (i) mature proinflammatory IL-1β and IL-18; and (ii) K^+^, causing an intracellular ion dyshomeostasis. Persistent K^+^ losses lead to inflammatory death or pyroptosis of the involved cells. In turn, products released from pyroptotic cells (e.g., ATP, mitochondrial DNA) boost inflammation further [18]. NLRP oligomerization, ASC recruitment, and caspase-1 nucleated polymerization/activation are irreversible processes developing in a self-inducing prion-like fashion and promoting canonical inflammasome signaling [19]. 

Moreover, via CARD, domain-assembled NLRP1, NLRP2, NLRP3, and AIM2 inflammasomes activate the NF-κB signaling pathway, which transcriptionally regulates the genes encoding for the various inflammasomes’ structural proteins [20]. Conversely, other NLRs, i.e., NLRC3, NLRP6, NLRP12, and NLRX1, impede the NF-κB pathway’s activation, thereby mitigating or quelling inflammation [21]. Indeed, these “*anti-inflammasomes*” are crucially necessary, as they stop the onset of chronic inflammatory diseases. Moreover, CARD-only proteins (COPs) and PYD-only proteins (POPs) also regulate inflammasome activity [22]. Furthermore, epigenetic mechanisms, e.g., noncoding RNA expression, CpG island DNA methylation, and histone post-translational changes, modulate inflammasome function [23].

We recently reviewed the multiple roles of the NLRP1, NLRP2, AIM-2, and NLRC4 inflammasomes in human and rodent brain diseases [24]. Our work showed that several inflammasomes can partake in brain neuroinflammatory processes. This enticed us to review in this work the mounting literature specifically concerning the NLRP3 inflammasome, its modulation by endogenous and exogenous and pharmacological and ethnopharmacological agents/extracts, its pathogenetic implications in acute and chronic brain diseases, and the therapeutic potential of its inhibition. Based on the results we highlight that disease-specific divergent mechanisms activate the brain’s NLRP3 in microglia/monocytes and other neural cell types. However, no proof is hitherto available that NLRP3 inhibition would be a human brain disease-modifying approach. Furthermore, no data have so far excluded the possible functional replacement of the inhibited NLRP3 by other concurrently activated inflammasomes. These facts led us to highlight that one of the causes of the persisting failures of human brain disease-related therapeutic attempts is the inadequate regard for its morpho-functional uniqueness based on the assumption that animal brain models are good enough. The consequent suggestion is to focus instead on human neural cell-based preclinical brain diseases models, which could drive etiological, pathogenetic, and therapeutic advances, including proper NLRP3 and other inflammasome regulation, and minimize failure risks concerning lead candidate drug testing in clinical trials. 

The following paragraphs will delve into the main advances concerning the NLRP3 inflammasome, followed by specific paragraphs about its role in most relevant brain diseases, a discussion of the results, and a conclusion. 

### 1.2. Brain NLRP3 Inflammasome

The inactive NLRP3 inflammasome (i.e., NLRP3-ASC or NOD-like receptor protein 3 (N-terminal PYD, central ATP-hydrolyzing NACHT (NAIP+CIITA+HET-E+TP1), and C-terminal LRR domains) molecules confine themselves to the endoplasmic reticulum (ER) membranes [25]. Upon activation, they bind adaptor ASC proteins by interacting with phosphatidylinositol-4-phosphate. ASC stabilizes the NLRP3•ASC complexes allowing their activation. Next, NLRP3•ASC complexes migrate to the perinuclear ER membranes and associated mitochondrial aggregates [9,26]. 

As monocytes/macrophages and microglia strongly express the NLRP3 inflammasome, the latter is involved in human brain diseases and is the most intensely studied and popular inflammasome. NLRP3 might be the “golden” therapeutic target of inflammatory morbidities, including neurodegenerative disorders (e.g., Alzheimer’s disease [AD]) [27,28,29]. In advanced age, the NLRP3 inflammasome also partakes in low-grade sterile yet chronic inflammation called “*inflammaging*”, driven by cell debris accumulating within tissues [30]. Moreover, NLRP3 gene mutations result in a spectrum of autoinflammatory diseases known as cryopyrin-associated periodic syndromes (CAPS) [31]. 

Table 1 lists the common brain NLRP3 inflammasome-activating diseases or agents.

#### 1.2.1. NLRP3 Inflammasome Priming and Canonical Activation

Importantly, human, and rodent brain cells of all types preferentially express distinct inflammasomes, e.g., NLRP1 the neurons, NLRP2 the astrocytes, and NLRP3 the microglia [123,124,125,126,127,128,129]. However, under both normal and pathological conditions, all the neural cell types express the NLRP3 inflammasome, albeit with differing intensities and regulatory mechanisms [27,64]. Young mice brains physiologically express basal levels of NLRP3 inflammasome activity to upkeep conditioning-induced neuronal plasticity and memory consolidation in the ventral hippocampus and basolateral amygdala [130]. Discordant opinions exist about inflammasomes’ roles in human brain diseases, as specific molecular lines of evidence are scanty [24,131,132]. 

Most studies have shown that NLRP3′s canonical activation requires two initiating signals. The “Signal 1” or “*priming step*” is an endocytosed PAMP or an endogenous DAMP/HAMP evoking the signaling from Toll-like receptor 4 (TLR-4) or a NOD-like receptor (NLR) or the tumor necrosis factor receptor (TNFR). Furthermore, signaling from G-protein-coupled receptors (GPCRs) can affect NLRP3 activity (see Box 1 for further details and references).

Box 1 **NLRP3 inflammasome regulation by G-protein coupled receptors (GPCRs).**

The six GPCRs families (A–F) include eight hundred entities. The fact that 34% of FDA-approved drugs target GPCRs proves their clinical importance. For space reasons here, we discuss only a few GPCRs. For further information, see [133].

  B1.1. *Calcium-Sensing Receptor (CaSR)*

  The extracellular domain (i.e., venus flytrap) of the ubiquitously expressed CaSR of family C GPCRs binds not only Ca^2+^, its orthosteric (type I) agonist, but also other mono-, bi-, and tri-valent cations, and various positively charged organic molecules, including polyamines, aminoglycoside antibiotics, and cationic peptides (e.g., amyloid-β [Aβ]) [134,135,136]. Moreover, CaSR’s 7TM (seven-pass transmembrane domain) binds allosteric (type II) ligands (e.g., aromatic L-α-amino acids) and positive allosteric modulators (PAMs i.e., calcimimetics) and negative allosteric modulators (NAMs i.e., calcilytics). Ligand-activated CaSR signaling by its intracellular domains is mediated by various G-proteins and scaffold proteins (e.g., β-arrestin, homer-1) and turns on or off several pathways involving various enzymes, ion channels, and transcription factors [133]. Acting as a calciostat sensing changes in [Ca^2+^]_e_, the CaSR regulates systemic [Ca^2+^]_e_ homeostasis via parathormone secretion, modulating gut Ca^2+^ absorption, bone Ca^2+^ storage/release, and renal Ca^2+^ excretion [137]. All types of neural cells express the CaSR, and those in AD-relevant hippocampus very intensely [138]. Importantly, besides [Ca^2+^]_e_ homeostasis, the CaSR physiologically regulates neural cell growth, differentiation, migration, synaptic plasticity, and neurotransmission [133]. Moreover, the CaSR acts as a DAMP/HAMP/PAMP sensor, as inflammatory diseases affecting various organs, brain included, activate CaSR signaling [27]. In turn, CaSR signaling activates the NLRP3 inflammasome via a surge in phospholipase C-mediated [Ca^2+^]_i_ and a concurrent fall in the NLRP3-inhibiting cAMP [31], as well as a proteolytic cleavage of crucial NLRP3 regulators [139]. Moreover, increasing cAMP levels via an adenylate cyclase (AC) activator (e.g., PGE2) or a covalently changed (e.g., dibutyryl-) cAMP or a phosphodiesterase (PDE) inhibitor blocking cAMP catabolism to 5′-AMP (e.g., theophylline or milrinone) promotes cAMP binding to NLRP3, which hinders its activation [26,31,140]. CaSR PAM cinacalcet activates NLRP3 inflammasome via ERK1/2 signaling [98]. Wang et al. [99] showed that in subarachnoid hemorrhage-model mice, CaSR’s expression surged in all CNS cell types. The CaSR agonist gadolinium trichloride (GdCI_3_) upregulated the levels of phosphorylated CaMKII, NLRP3 inflammasome expression, active caspase-1, and mature IL-1β. Conversely, CaSR NAM NPS-2143 and CAMKII inhibitor KN-93 mitigated all CaSR signaling detrimental effects. Hence, CaSR signaling advanced the first stages of acute brain injury, and Aβ•CaSR signaling could drive human AD onset/progression [141].

  B1.2. *G-Protein-Coupled Class C Group 6 Receptor A (GPC6RA)*

  Alum has been and still is in use as an adjuvant in human vaccines. Alum’s mechanism of action remained obscure until Quandt et al. [50] proved that in vitro and in vivo alum induced NLRP3 inflammasome activation via GPRC6A receptor signaling. GPC6RA, of the GPCR Family C Group 6, senses cations (e.g., Ca^2+^), osteocalcin, L-α-amino acids, and testosterone. GPC6RA signaling partakes via MAPK and mTORC1 in prostatic carcinoma progression [51,142,143,144,145] and might contribute to the angiotensin II-driven hypertensive neuroinflammation promoted by 6β-hydroxytestosterone in male mice [146].

  B1.3. *G protein-coupled estrogen receptors (GPERs)*

  GPER1 and GPER30 are seven-pass transmembrane orphan receptors that rapidly mediate non-genomic estrogen-related kinase signaling. GPER signals prevented hippocampal neuron death due to transient global cerebral ischemia via a remarkable elevation of the endogenous interleukin-1 receptor antagonist (IL-1Ra), which suppresses the pro-inflammatory effects of IL-1β. GPER activation heightened the hippocampal levels of phosphorylated CREB (i.e., cAMP response element-binding) transcription factor, which promotes IL-1Ra expression. The G36 antagonist reversed GPER’s neuroprotective effects, proving their specificity [147].

  Clearly, CaSR, GPC6RA, and GPERs are PRRs whose roles in neuroinflammation are worthy of further investigation.

Signal 1 involves both translational and post-translational pathways linked to IFNR, PKA, MAPK, mTOR, complement proteins, AMPK/autophagy, IRAK1, TRIF (TIR[Toll/IL-1 receptor/resistance protein]-domain-containing adapter-inducing IFN-β), and NLRP3’s de-ubiquination by BRCC3 (BRCA1/BRCA2-Containing Complex Subunit 3), a Lys^63^-specific de-ubiquitinase. These pathways converge toward NF-κB pathway’s activation, which mediates the genetic transcription of NLRP3, ASC, pro-caspase-1, pro-IL-1β, and pro-IL-18 [148,149,150,151,152]. The contours of “Signal 2” or the “*activation step*” of the NLRP3 inflammasome are less defined. A summary list of Signal 2 includes exogenous dead cell-released ATP, which is a ligand of purinergic receptors (see Box 2 for further details and references); cathepsin B released from destabilized lysosomes; phagocytosed protein polymers; reactive oxygen species (ROS); cardiolipin; oxidized mitochondrial DNA [112,114]; K^+^ efflux or Ca^2+^ influx, independently of each other [153]; and cyclic AMP (cAMP) downregulation [154]. Importantly, also contact sites between mitochondria and ER membranes favor NLRP3 activation. ER-stress signal-released mitochondrial proteins, ER-released Ca^2+^ surges, lipid perturbations, and cholesterol trafficking critically partake in NLRP3 activation [155]. Moreover, a surge in extracellular Ca^2+^ ([Ca^2+^]_e_) triggers NLRP3 activation in monocytes [156]. Thus, [Ca^2+^]_i_ increases might be the signal shared by all the stimuli [155] and/or the final common NLRP3-activating pathway [157,158].

Box 2 **Brain purinergic receptors.**

CNS neural cells express diverse types of purinergic receptors, i.e., P1, for adenosine G protein-coupled receptors; P2X, for ATP-gated ion channels; and P2Y, for G protein-coupled receptors. Importantly, the intra-brain accumulation of Aβs induces the damaged neural cells to release ATP into the extracellular matrix (ECM). Exogenous ATP and the agonist 4-benzoyl-ATP (BzATP) activate the signaling from P2X_7_ purinergic receptors expressed by neural cells. The upshots are an increased synthesis and release of pro-inflammatory cytokines and chemokines, and a decline in the α-secretase activity, causing a plunge in the extracellular shedding of the neurotrophic and neuroprotective soluble amyloid precursor protein (APP)-α. Yet, various (e.g., mechanical) stressing factors awaken the signaling of P2X_7_ receptors, making the cells release their endogenous ATP through connexin 43 and pannexin hemichannels (i.e., “pathological pores”) [159]. The results are the activation of the NF-κB axis and of the NLRP3•ASC•caspase-1 and IL-1β pathways in both the astrocytes and microglia, triggering the sterile neuroinflammation proper of AD within the brain and of glaucoma within the retina [57,160].

  Moreover, the P2X_7_ receptor agonist BzATP also elicits the release of various cytokines from the retinal ganglion neurons, i.e., IL-3 (in the presence of extracellular Ca^2+^); IL-4; IL-10; IL-1Ra; TNF-α; MIG/CXCL9 (or monokine induced by IFN-γ/chemokine [C–X–C motif] ligand 9); VEGF; GM-CSF; MIP (macrophage inflammatory protein); CCL20 (or chemokine [C–C motif] ligand 20); and L-selectin, which altogether exert neuroprotective effects [161]. P2X_7_ receptor stimulation also upregulates IL-6 release from the retinal astrocytes and neurons [162]. In microglial cells, P2X_7_ receptors modulate the phagocytosis of exogenous debris in the absence of any ligand. However, signals from ligand-bound P2X_7_ alter lysosome function, causing the cathepsin B-mediated NLRP3 inflammasome activation that a cathepsin B-blocker, CA-074, instead hinders [163].

  P2X_7_^−/−^ (KO), P2X_7_ antagonists, such as Brilliant Blue G (BBG), A438079, A839977 and A740003, and the NF-κB inhibitor Bay 11-7082 blocked the effects elicited by purinergic receptors signaling. However, P2X_7_-specific antagonists blocked only the purinergic receptor-dependent secretion of IL-6 and CCL2 but not TNF-α’s release from microglia. These results revealed the differential regulation of the microglial secretion of such cytokines [164]. By contrast, the ATP-activated signaling from the P2Y_2_ purinergic receptor exerted P2X_7_-opposite, i.e., anti-inflammatory, and neuroprotective effects [165,166].

Nuclear receptors too control the NLRP3 inflammasome [167]. Thus, various positive and negative signaling pathways strictly regulate NLRP3’s activation to prevent any harm while preserving the host tissues’ homeostasis [168]. Various kinases, ubiquitin ligases, a de-ubiquitinase, and other enzymes crucially control both NLRP3’s activation and function termination via ad hoc post-translational modifications of its protein components [169]. As an example, Bruton’s tyrosine kinase (BTK) directly and positively regulates the NLRP3 inflammasome, which might have therapeutic implications [170]. Usually, sterile, and slow-acting DAMPs/HAMPs elicit weaker NLRP3 inflammasome responses than infectious PAMPS do [171]. Finally, inflammasome-interested scientists should note that species-related differences in animal models can crucially affect their results [172].

#### 1.2.2. Noncanonical NLRP3 Activation

Hitherto, we have discussed NLRP3’s “canonical activation”, a concept valid also for NLRP1, NLRC4, and AIM2 inflammasomes. The more recently discovered “noncanonical activation” of inflammasomes is worth mentioning too. Concerning microglia’s NLRP3, the noncanonical process involves the activation of caspase-11 and caspase-8 in mice and of caspase-4 and caspase-5 in humans [173,174,175]. These caspases behave as cytosolic sensors that directly bind and are activated by the lipopolysaccharide (LPS) of Gram-negative bacteria. This drives the secretion of mature IL-1β and IL-18. Additionally, the active caspases detach N-terminal fragments from the GSDMD/GsdmD proteins, which form transmembrane pores promoting K^+^ efflux and thus causing both NLRP3’s canonical activation and neurons’ pyroptosis [176,177,178,179]. 

The HMGB1 (high mobility group box 1 protein)/caspase-8 pathway is an added mechanism of noncanonical NLRP3 activation proper of eye glaucoma. An acutely elevated intraocular pressure intensifies HMGB1’s signaling, which activates the NLRP3 inflammasome by canonical and noncanonical (via caspase-8) mechanisms, producing higher amounts of mature IL-1β within the ischemic retinal tissue and thereby advancing neuroinflammation [59].

### 1.3. Brain NLRP3 Inflammasome’s Modulation by RNAs

Cells express manifold kinds (ribosomal, messenger, and noncoding) of RNAs, which control most of their functions. Long noncoding (Lnc) RNAs have more than 200 base pairs but encode no or few proteins. However, LncRNAs importantly affect body development, cell differentiation, metabolism, autoimmunity, and immune function, and hence NLRP3 inflammasome activity [180,181]. MicroRNAs (or miRs) are ubiquitous 22-nucleotide-long single-stranded RNAs that post-transcriptionally control gene expression by silencing mRNAs via complementary base-pairing [182]. Notably, miRs abound (>2300 types) inside mammalian cells and are released via extracellular vesicles (EVs) or exosomes (Exos) into cerebrospinal fluid and blood. Circulating miRs are under investigation as biomarkers in various diseases and in the distinct stages of each illness. According to ongoing circumstances, distinct miRs promote or inhibit NLRP3 inflammasome activation.

Among noncoding RNAs, Alu-derived RNAs deserve a brief mention. They result from the transcription of primate-specific transposable “Alu elements” by small interspersed nuclear elements (SINEs). Alu-RNAs are plentiful, involving >10% of the human genome, with 102 to 103 copies released into the cytosol of each cell. Alu-RNAs regulate gene expression by binding and inhibiting RNA polymerase II (P2). Alu-RNAs accumulate in the brains of patients with dementia or sporadic Creutzfeldt–Jacob’s disease (CJD), in which they drive neuroinflammation and neuron demise [183]. P3-transcribed Alu-RNAs (P3Alus) may advance NLRP3 inflammasome-driven neuroinflammation/neurodegeneration disorders, AD included [184]. Hence P3Alus may be therapeutic targets for such ailments. Later studies revealed that Alu-RNAs processing rates are elevated in mouse and human AD brains, tightly correlating with the up-regulated expression of HSF1 (heat shock transcription factor 1), a crucial stress response factor. The increased Alu-RNAs processing rates would fix into active mode the HSF1/Alu-RNA/stress response/cell death-promoting genes (e.g., p53) axis in AD patients [185,186].

This topic is bound to undergo further developments in regard not only to LncRNAs, miRs, and Alu-RNAs, but also to the recently discovered circular RNAs [187].

Table 2 reports details about LncRNAs/miRs and NLRP3 interactions. 

### 1.4. Brain NLRP3 Inflammasome’s Modulation by Extracellular Vesicles (EVs) and Exosomes (Exos)

EVs partake in neuroinflammation-promoting intercellular signaling. Exos are a class of EVs extruded by any cell type. Exos originate in multivesicular bodies, have sizes of 30–100 nm, and bear specific tetraspanin family markers on their membranes. Exos enclose and convey high numbers of functional proteins, lipids, and regulatory RNAs, which affect recipient cells’ metabolic activities, proliferation, or death. Hence, nerve cell-released Exos can act as “either friends or foes” to neurons depending upon their cargoes (e.g., growth factors or Aβs or p-Taues) [60,208] (v. Table 2). In a model of microglial BV-2 cells, pyroptosis induced by O_2_-glucose deprivation/reperfusion (OGD/R), human mesenchymal stem cells (MSC)-released Exos (huMSC-Exos) increased FOXO3a gene expression, thereby enhancing mitophagy while reducing the levels of NLRP3; cleaved caspase-1, IL-1β, IL-18; GsdmD-N fragments; and pyroptosis. Hence, huMSC-Exos might mitigate human neurons’ OGD/R-induced pyroptosis [209]. Consistently, bone marrow MSC-derived Exos (BMMSC-Exos) intravenously injected 2 h after middle cerebral artery occlusion (MCAO) decreased brain infarct volume, NLRP3 protein expression, and neuron pyroptosis. Moreover, BMMSC-Exos administration shifted the ischemia-induced microglial proinflammatory M1 phenotype to the homeostatic M2 [210].

Cui et al. [197] reported that Exos released from hypoxia-preconditioned MSCs (MSC-Exos) downregulated TNF-α and IL-1β, hindered NF-κB and STAT3 (signal transducer and activator of transcription 3) activation, and decreased Aβ peptides levels and senile Aβ plaques, while upregulating anti-inflammatory IL-4 and IL-10, and exo-miR-21, which improved memory and learning in APP/PS1 AD-model mice. In another study, Cui et al. [211] used the CNS-specific rabies viral glycoprotein (RVG) to target intravenously infused Exos released from MSCs (MSC-RVG-Exos) to the cerebral cortex and hippocampi of transgenic APP/PS1 AD-model mice. MSC-RVG-Exos downregulated IL-1β, TNF-α, and IL-6, while upregulating anti-inflammatory IL-10, IL-4, and IL-13.

In summary, the available evidence about EVs’ and Exos’ beneficial or harmful roles in NLRP3-mediated neuroinflammation is still scanty. A further limitation is that most studies focused on the RNAs conveyed by EVs or Exos. However, EVs or Exos also transport high numbers of different proteins that either promote or hinder neuroinflammation. In fact, Exos from Aβ_25–35_-exposed human cortical astrocytes conveyed significantly increased amounts of p-Taues [212], while Exos from human AD brains transported Aβ oligomers [213].

### 1.5. Other Brain NLRP3 Inflammasome Regulators

Under any situation, complex sets of endogenous factors control or restrain NLRP3 inflammasome assembly and/or function, trying to reestablish and/or upkeep tissue homeostasis. Zhang et al. [214] strengthened the relevance of the NLRP3 concept by proving that NLRP3 gene knockout or pharmacological blockage improved the course of various inflammatory diseases modeled in rodents. Hereafter we mention relevant NLRP3 regulators.

The zinc-finger protein A20, i.e., TNFAIP3 (TNF-α-induced protein 3), has two functions: it blocks apoptosis and crucially controls microglia function by inhibiting NF-κB activation in CNS physiological and pathological conditions. A20 knockout led to NLRP3 inflammasome’s hyperactivation, increasing mature IL-1β secretion and neuroinflammation intensity [215]. 

Additionally, CD40 (i.e., cluster of differentiation 40) protein, a member of the TNFR superfamily, negatively affected the ATP•TLR4-signaling-mediated NLRP3 inflammasome’s activation in microglia. Therefore, it regulated microglia’s inflammation-initiating Th17 response triggered by DAMP-induced brain injuries [216]. 

Mitsugumin-53 (i.e., TRIM-72 or tripartite motif 72) protein partook in damaged plasma membranes repair and inhibited the NLRP3/caspase1/IL-1β pathway and TNF-α expression, thus mitigating neuroinflammation [217]. Conversely, the TRIM-21 protein promoted microglia’s pro-inflammatory M1 phenotype polarization that TRIM-21’s knockout reversed [218]. 

Osteopontin is a highly phosphorylated ECM sialoprotein expressed during the subacute phase following cerebral infarction. It stimulated microglia’s chemotaxis while preventing NLRP3’s activation and its sequels [52].

Worth mentioning here is PKR (i.e., protein kinase RNA-activated), a multirole serine–threonine kinase controlling mRNA transcription/translation, protein synthesis, cell proliferation, apoptosis, and brain function, in addition to shielding cells from viral infections. A dysfunctional PKR partook in cancer and neuroinflammation [219]. Moreover, by using wild-type and PKR^−/−^ mouse macrophages, Lu et al. [220] showed that PKR needed to physically interact with NLRP3, NLRC4, and AIM-2 inflammasomes to activate them. However, using LPS-treated PKR^−/−^ bone marrow-derived macrophages isolated from different mouse strains, He et al. [221] reported that following stimuli activating NLRP3, NLRC4, and AIM2 inflammasomes’ PKR activity was critical for nitric oxide synthase-2 (NOS-2) induction, yet dispensable for pro-IL-1β and pro-IL-18 cleavage by caspase-1 [172]. Altogether the divergent results of Lu et al. [220] and Healy et al. [172] show that the animal species or strains investigated do significantly affect the kind of mechanisms activating or inactivating the NLRP3 and other inflammasomes. This adds a remarkable degree of complexity to the topic and stresses the importance of investigating corresponding mechanisms in human neural cells models.

### 1.6. Brain NLRP3 Inflammasome Inhibitors

Inhibiting the NLRP3 inflammasomes has been a tantalizing enterprise given its potential therapeutic applications in brain diseases. Table 3 lists the reported NLRP3 inhibitors, of which MCC950 is the most popular one in experimental works [222], although it failed in a clinical trial due to off-target toxic effects. 

### 1.7. Brain NLRP3 Downregulation by Officinal Plant Agents/Herbal Extracts

Since time immemorial, plants were and still are the source of drugs helping human ailments. Although extracts of plant body portions are still in use in Traditional Chinese Medicine (TCM), the current more scientific attitude is to find the specific compound(s) of potential therapeutic use. Table 4 reports the most relevant agents and herbal extracts of interest regarding the brain NLRP3 inflammasome. 

It is worth noting that save for ginsenoids, artemisinin, and artesunate, all the other hitherto-reported therapeutically promising plant agents/herbal extracts still need in-depth preclinical studies and well conducted clinical trials prior to becoming FDA-approved drugs. On the other hand, altogether the above-listed agents/extracts represent a treasure trove of future therapeutic assets.

## 2. NLRP3 Inflammasome in Brain Acute Injuries

Glial NLRP3’s role is controversial in HI/OGD (oxygen–glucose deprivation)-model animals. Denes et al. [336] reported that plasma IL-18 levels and brain infarction volume were alike in both wild-type and NLRP3-shRNA-silenced mice. Therefore, NLRP3’s downregulation was not as neuroprotective as expected because other inflammasomes took over and functioned in NLRP3’s stead. In fact, after shRNA-induced NLRP3 depletion, OGD significantly increased AIM2 inflammasome’s expression while NLRC4’s expression did not change in BV-2 microglial cells. 

Conversely, Yang et al. [337] showed that in newborn mouse astrocytes HI and OGD activated TRPV1 (transient receptor potential vanilloid 1), a non-selective cation channel of the TRP family. Next, the TRPV1 signaling drove the JAK2-STAT3 pathway, which mediated NLRP3 inflammasome’s activation and increased IL-1β levels. Notably, in HI- and OGD-exposed TRPV1^−/−^ mouse astrocytes, JAK2 and STAT3 activation and IL-1β upregulation were less intense. Interestingly, this study revealed different cell type-related timings of NLRP3 activation elicited by HI/OGD. In newborn mouse astrocytes of the hippocampus, striatum, and thalamic habenula, NLRP3’s activity increased by 3 h, while in microglia it was insignificant at 3 h but increased remarkably by 72 h. Then again, Schölwer et al. [338] showed that OGD completely inactivated phagocytic activity in wild-type BV-2 cells, while HI restored phagocytic activity in NLRP3-shRNA-depleted BV-2 cells. Therefore, the authors posited that NLRP3 plays a minor replaceable role in the OGD-elicited neuroinflammation, at least in microglia. Conversely, an anti-inflammatory pleiotropic cytokine, IL-10, hindered NLRP3 activation in microglia by increasing STAT-3’s function, which stifled the transcription/translation of pro-IL-1β and mature IL-1β production [339].

Relevant to this topic is IL-33, another IL-1 family member playing major pleiotropic roles in normal and pathological conditions [340]. In neonatal mouse astrocytes, IL-33 expression markedly increased by 24 h after a cerebral HI episode. Exogenously administered IL-33 did mitigate brain infarction volume by one week after the HI event. Astrocytes’ basal expression of ST2 (or suppressor of tumorigenesis 2), the IL-33 receptor, was intense and after HI exposure increased further. Conversely, a ST2 shortfall worsened the HI-elicited brain infarction. The IL-33•ST2 signaling-activated pathways mitigated astrocytes’ HI-elicited neuroinflammatory response and apoptosis. Moreover, in vitro IL-33-treated murine astrocytes released neurotrophic factors, which protected HI- and OGD-exposed neurons’ viability [341]. Besides, administering IL-33 plus MCC950 and antimalarial drugs improved the outcome in a model of murine cerebral malaria [342] in which the *Plasmodium falciparum* overgrew inside the cortical capillaries, diffusely obstructing blood flow.

Franke et al. [36] showed that following stroke’s onset, the early up-regulation of the NLRP3 inflammasome occurred in neurons, glia, and vascular endothelia, leading to blood–brain barrier (BBB) breakdown. Consistently, NLRP3 inhibition hindered endothelial pyroptosis induced by the thrombolytic agent rt-PA (or tissue plasminogen activator), thus preserving the BBB’s integrity [11]. Similarly, NLRP3-inhibitor MCC950 protected brain endothelial cells from rt-PA’s toxic effects in an in vitro HI-exposed BBB model [343]. Additionally, NLRP3′s knockout alleviated the NF-κB pathway-mediated brain damage in a middle cerebral artery occlusion (MCAO)-induced focal ischemia mouse model [344]. Moreover, lithium (Li^+^), the archetypal mood stabilizer, also impeded HI/R-induced NLRP3 inflammasome activation, and by stimulating STAT3’s function improved motor behavior, cognition, and depression [345]. 

**Figure 1:**
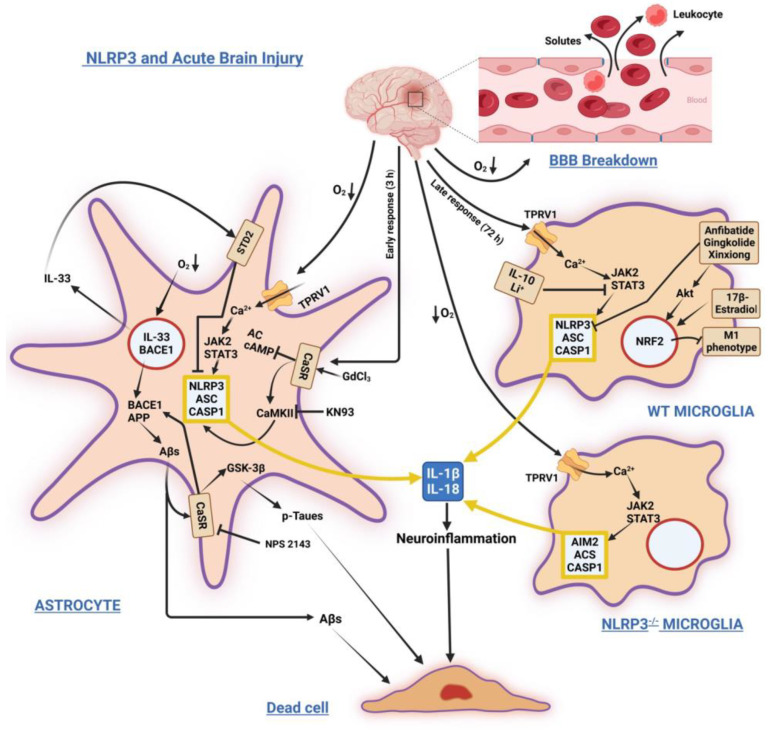
Schematic illustration of stressors and factors inducing/modulating NLRP3 inflammasome’s activation and its sequels in astrocytes and microglia under acute injuries due to hypoxic ischemia, stroke, and hemorrhage. Left: Astrocyte’s prompt response. Acute O_2_ tension fall activates Ca^2+^ influx through TPRV1 channels, triggering the JAK2/STAT3 axis and NLRP3 inflammasome activation. It also increases BACE1 and IL-33 gene expression. Over-released IL-33 binds its STD2 receptor, whose signaling mitigates NLRP3 activity. Later, BACE1 increased activity overproduces Aβs. Extracellularly released excess Aβs bind and activate CaSR signaling, which contributes to NLRP3 inflammasome activation by reducing cAMP levels and activating CaMKII. Aβ•CaSR signaling also increases BACE1 and GSK-3β activities, driving the over production of Aβs from APP and p-Taues, which are both intracellularly accumulated and extracellularly released. CaSR NAM (Calcilytic) NPS2143 and CaMKII inhibitor KN93 suppress Aβs•CaSR signaling noxious effects (see for more details Box 1). Top right: Late wild-type microglia response. The NLRP3 activation is blocked by various agents, which activate via Akt the expression of NRF2 transcription factor. NRF2 activity reduces the M1 (proinflammatory) fraction of microglia. Bottom right: In a model of NLRP3 full-knockout microglia Ca^2+^ influx activates in NLRP3 stead the AIM2 inflammasome’s signaling, the upshot being the same, i.e., the overproduction/release of IL-1β and IL-18 [336]. A yellow frame encloses the assembled inflammasomes, while nuclear envelopes are orange colored. Abbreviations: Aβs = amyloid-β peptides; AC = adenylyl cyclase; AIM2 = absent in melanoma 2 inflammasome; Akt = protein kinase B; APP = amyloid precursor protein; ASC = apoptosis-associated speck-like protein endowed with a caspase recruitment domain or CARD; BACE1 = β-secretase; BBB = blood-brain barrier; cAMP = 3′,5′-cyclic adenosine monophosphate; CASP1 = caspase-1; CaMKII = Ca^2+^/calmodulin-dependent protein kinase II; CaSR, calcium-sensing receptor; GdCl_3_ = gadolinium chloride; GSK-3β = glycogen synthase kinase-3β; JAK2 = Janus kinase 2; KN93 = N-[2-[[[(E)-3-(4-chlorophenyl)prop-2-enyl]-methylamino]methyl]phenyl]-N-(2-hydroxyethyl)-4-methoxybenzenesulfon-amide; NPS-2143 = 2-chloro-6-[(2R)-2-hydroxy-3-[(2-methyl-1-naphthalen-2-ylpropan-2-yl)amino]-propoxy]-benzonitrile; p-Taues = hyperphosphorylated Tau proteins; STAT3 = signal transducer and activator of transcription 3); STD2 = suppression of tumorigenicity 2 (receptor); TPRV1 = vanilloid type 1 receptor/channel; WT = wild-type. ↓O_2_ = decrease in oxygen tension. The other arrows show the sequences of molecular events induced by stressors and factors. ⊥ = inhibition.

Figure 1 sums up the main signaling pathways involving NLRP3 in acute brain injuries.

Finally, electroacupuncture (EA) exerted analgesic effects by suppressing NLRP3 inflammasome function in the spinal dorsal horn of mice [346]. Moreover, EA at the skull’s *Shenting* (DU24) and *Baihui* (DU20) acupoints attenuated cognitive impairment in rats with brain HI/R injury by regulating endogenous melatonin secretion through alkylamine N-acetyltransferase synthesis in the epiphysis. Next, melatonin acted neuroprotectively by blocking NLRP3 activation via upregulating mitophagy-associated proteins [347].

In conclusion, given the consistent risk that an acute brain injury triggers a chronic neurodegenerative disease entailing a lethal outcome, the therapeutic mitigation or better suppression of neuroinflammation within a brief time lag following the harmful event constitutes a quite valid target to be pursued.

## 3. NLRP3 Inflammasome in Chronic Neurodegenerative Disease

### 3.1. Alzheimer’s Disease (AD)

AD is the most prevalent human dementia. Under healthy conditions, the NLRP3 inflammasome is inactive in microglia and astrocytes. Halle et al. [348] first showed that Aβ fibrils—AD’s main drivers together with p-Taues and neuroinflammation—activate microglia’s NLRP3 inflammasome in APP/PS1 AD-model mice. After phagocytosis by primary mouse microglia, Aβ_1–42_ fibrils damaged the lysosomes, which released cathepsin B, activating the NLRP3 (previously named NALP3) inflammasome and IL-1β, TNF-α, and nitric oxide (NO) overproduction. In turn, the activated NLRP3 inflammasome intensified AD neuropathology in vivo well before Aβs senile plaques appeared [348,349,350]. Heneka et al. [349] also showed that NLRP3 inflammasome’s downregulation shifted microglia’s polarization toward the homeostatic M2 phenotype, concurrently depleting the brain’s Aβs load. Hence, they posited that NLRP3 inflammasome activation remarkably partook in the microglia-mediated persistent neuroinflammation observed in AD-model mice. Consequently, NLRP3’s inhibition would be a novel anti-AD therapeutic approach. Consistently, NLRP3-blocking dihydromyricetin [239] or MCC950 [222] promoted the brain’s Aβs clearance, increased hippocampal and cortical M2 microglia fractions, and improved memory and cognition in APP/PS1 mice.

Astrocytes are by far the most abundant cell type populating the brain. Hence, any astrocytes’ contributions to neuroinflammation are quite relevant to the progression/outcomes of neurodegenerative diseases. ASC is an adaptor protein forming stable NLRP3•ASC complexes acting as inflammasomal activation hubs. Studies using ASC^+/−^ or ASC^−/−^ 5xFAD newborn mice proved that Aβs do activate astrocytes’ inflammasome(s). In ASC^+/−^ mice, NLRP3 inflammasome activity was downregulated; concurrently, an upregulated MIP-1α/CCL3 release increased Aβs phagocytosis by lipopolysaccharide (LPS)-primed primary newborn 5xFAD mouse astrocytes. Moreover, in 7–8-month-old ASC^+/−^ 5xFAD mice, Aβs’ brain load downfall correlated with upregulated CCL3 gene expression and improved spatial reference memory [351,352]. Furthermore, ASC moieties released from pyroptotic neurons bound extracellular Aβs and cross-seeded Aβs’ increase, promoting NLRP3 inflammasome’s activation, neuronal pyroptosis, and neuroinflammation. In turn, these effects increased ASC’s available moieties, triggering a self-sustaining feedforward vicious loop while undermining microglial Aβs clearance [353].

Murphy et al. [354] showed that exposure to Aβs increased cytosolic cathepsin B’s protease activity, which drove NLRP3 inflammasome’s activation and IL-1β over release from wild-type rat primary glial cultures. Consistently, the endogenous protease inhibitor α1-antitrypsin (A1AT) reduced Aβ_1–42_-elicited NLRP3’s activation and its sequels in primary cortical astrocytes from BALB/c mice [128,222].

More recent investigations using rodent astrocytes confirmed that exposure to Aβ_1–42_ or LPS inhibited the autophagy/lysosome function while activating the NLRP3/ASC/caspase-1/IL-1β pathway. However, the administration of rapamycin or 17β-estradiol (E2) or progesterone rescued autophagic activity while curbing the Aβ_1–42_- and LPS-activated NLRP3/caspase-/IL-1β pathway in the astrocytes. By contrast, 3-methyladenine, a specific autophagy inhibitor, blocked progesterone’s neuroprotective effects and drove astrocytes’ NLRP3 inflammasome activation and neuroinflammation [355,356].

Here, a mention is in order about the inducible thioredoxin-interacting protein (TXNIP), which partakes in oxidative stress and regulates thioredoxin (TRX), another redox controller. Both the unfolded protein response (UPR) and ER stress also activate TXNIP. Concurrently, UPR activates the IRE-1α (or inositol requiring enzyme-1α) stress sensor pathway, which in turn further increases TXNIP’s amounts susceptible of activation [357]. Importantly, TXNIP’s function is essential for the increased expression and activation of NLRP3’s inflammatory cascade, both in the aging-associated chronic inflammaging, which goes along with senile cognitive decline, and in the hippocampal neurons and microglia of AD brains [66,67,358]. In rodent models of AD, Aβ_1–42_ drove NLRP3 activation and oxidative damage via the formation of TXNIP•Keap1 (Kelch-like ECH-associated protein-1)•NRF2 (nuclear factor erythroid 2-related factor 2) complexes. Exposure to HJ105 or HJ22, both piperine derivatives, or 9-(NXPZ-2) or maxacalcitol, an active vitamin D analogue, directly inhibited the formation of Keap1•NRF2 complexes, upregulated NRF2’s nuclear expression, hindered TXNIP-mediated NLRP3 inflammasome activation, and blocked Aβ_1–42_ and oxidative stress noxious effects [359,360,361,362]. 

**Figure 2:**
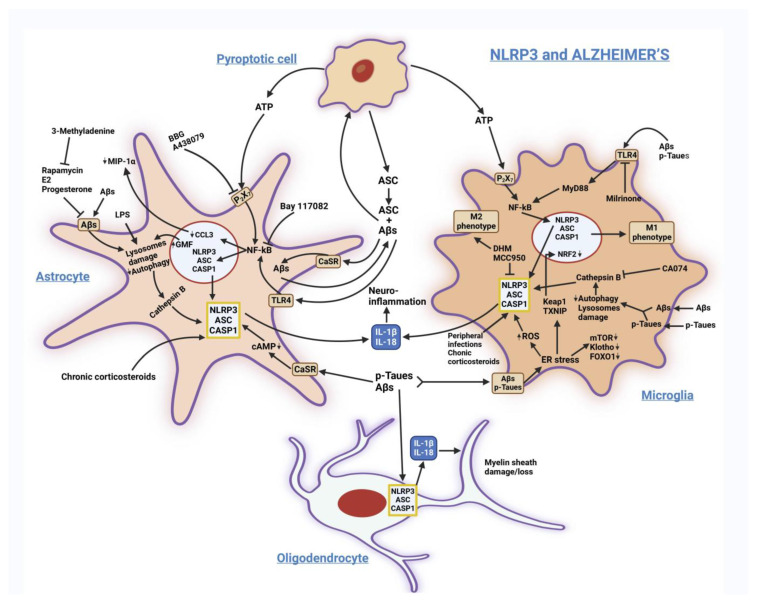
Schematic depiction of stressors and factors inducing/modulating glial cell NLRP3 inflammasome activation and its consequences in AD. Exogenous Aβs, p-Taues, ATP, ASC, IL-1β, and IL-18 interact with cell surface receptors, including CaSR (see Box 1), TL-4, and P2X_7_ (see Box 2), or are endocytosed to activate NF-κB and NLRP3 inflammasome signaling. They also induce ER stress, release Cathepsin B from damaged lysosomes, and block autophagy, while over-releasing further amounts of Aβs, p-Taues, and inflammatory cytokines. Altogether, they damage myelin sheaths and cause M1 microglial phenotype polarization and neuron and oligodendrocyte pyroptotic death. NLRP3 and receptor inhibitors mitigate the just-mentioned noxious effects. Additionally, the CaSR NAM NPS-2143 blocks Aβs, p-Taues, and IL-6 over production and release and reactivates autophagy (not shown; [181,199,354]). Regarding the roles of other-than-NLRP3 inflammasomes, see [24]. A yellow frame encloses the assembled NLRP3 inflammasomes, while nuclear envelopes are orange-colored. Abbreviations: A438079 = 3-[[5-(2,3-dichlorophenyl)tetrazol-1-yl]methyl]pyridine; Aβs = amyloid-β peptides; ASC = apoptosis-associated speck-like protein endowed with a CARD; Bay117082 = (E)-3-(4-methylphenyl)sulfonylprop-2-enenitrile; BBB = blood-brain barrier; BBG = brilliant blue G; cAMP, 3′,5′-cyclic adenosine monophosphate; CA074 = CAS 134448-10-5; CASP1 = caspase-1; CaSR = calcium-sensing receptor; CCL3 = gene encoding MIP-1α chemokine; DHM = dihydromyricetin; E2 = estradiol; FOXO1 = forkhead box protein O1; GMF, glia maturation factor; JAK2 = Janus kinase 2; Keap1 = Kelch-like ECH-associated protein 1; KN93 = N-[2-[[[(E)-3-(4-chlorophenyl)prop-2-enyl]-methylamino]methyl]-phenyl]-N-(2-hydroxyethyl)-4-methoxybenzenesulfon-amide; LPS = bacterial lipopolysaccharide; MCC950, CAS 210826-40-7; MIP-1α = monocyte chemoattractant protein-1α; mTOR = mammalian target of rapamycin; MyD88 = myeloid differentiation primary response 88; NF-κB = nuclear factor κB; P2X_7_ = purinergic receptor; p-Taues = hyperphosphorylated Tau proteins; STAT3 = signal transducer and activator of transcription 3; TLR-4 = Toll-like receptor 4; TPRV1, vanilloid type 1 receptor/channel; TXNIP = thioredoxin interacting protein. The small arrows close to a name indicate (↓) decrease, or (↑) increase in levels. ⊥ = inhibition. The other arrows show the sequences of molecular events induced by stressors and factors.

Figure 2 sums up the main signaling pathways involving NLRP3 in AD.

Notably, ER stress concurs with the depletion of the anti-aging and cognition-enhancing Klotho, FOXO-1, and mTOR proteins. Moreover, proteins partaking in ER stress development—such as BiP (binding immunoglobulin protein), eIF-2α (eukaryotic initiation factor-2α), and CHOP (C/EBP homology protein)—showed heightened levels of expression in the hippocampi of AD brains. Therefore, altogether TXNIP could link the chronic increases in glucocorticoids elicited by a persistent ER stress with AD’s enduring NLRP3 activation and neuroinflammation [67,363]. 

A newly identified gene associated with the risk of AD is *TREML2* (triggering receptor expressed on myeloid cell-like 2), a protein expressed by microglia [364,365]. TREML2 protein expression levels rise along with AD progression in vivo [366] and after LPS stimulation in primary microglia in vitro, both proving TREML2 involvement in microglia-induced neuroinflammation [367]. Then again, Wang et al. [368] showed that LPS stimulation or lentivirus-mediated TREML2 overexpression remarkably upregulated NLRP3 inflammasome activation; IL-1β, IL-6, and TNF-α secretion; and proinflammatory M1-type polarization in microglia of APP/PS1 AD-model mice. Therefore, TREML2 inhibition would be a novel anti-AD therapeutic approach.

Two studies showed that caspase-1-mediated overproduction of IL-1β occurred in brain samples from mild cognitive impairment (MCI) and fully symptomatic AD patients. Hence, in both groups, microglial NLRP3 inflammasome activation advanced AD’s persistent neuroinflammation [140,348]. Sokolowska et al. [140] also showed that phagocytosed Aβ_1–42_ fibrils damaged human macrophages’ lysosomes, which released cathepsin B into the cytosol, triggering the NLRP3•ASC•caspase-1 inflammasome’s oligomerization and activation. Moreover, studies conducted on brain tissue samples from AD patients that had died because of intercurrent systemic infections and APP/PS1 AD-model mice revealed that any added proinflammatory insults intensified NLRP3 inflammasome’s assembly/activation and IL-1β, IL-6, and various chemokines release from microglia, astrocytes, and neurons while increasing the brain’s Aβs and p-Taues load. Hence, any concurring etiologic factor could worsen neuroinflammation and hasten AD progression in humans [71,369,370]. 

Saresella et al. [371] reported the occurrence of a significantly upregulated expression of mRNAs encoding for NLRP1; NLRP3; ASC/PYCARD; caspase-1, -5, and -8; pro-IL-1β; and pro-IL-18 in monocytes isolated from MCI or late-stage AD patients. However, both NLRP1 and NLRP3 inflammasomes functioned only in late-stage AD monocytes. Conversely, ASC/PYCARD and caspase-1 expression was normal in early MCI monocytes in which assembled/functional inflammasomes were missing. Hence, concurrently activated NLRP1 and NLRP3 inflammasomes aggravated neuroinflammation only in late AD. 

Interestingly, in subjects with autistic spectrum disorders (ASD), Saresella et al. [131] found that both AIM2 and NLRP3 inflammasomes were active, overproducing IL-1β and IL-18. Simultaneously, there occurred an upregulation of the innate immunity suppressor IL-37, a decline of anti-inflammatory IL-33, and a rise in IFABP (intestinal fatty acid-binding protein—an altered gut permeability index). Therefore, multiple inflammasomes are active in both AD and ASD. 

Immunohistochemical studies conducted on samples of temporal cerebral cortex of AD brains showed that the increased expression of NLRP3 inflammasome’s constituents, including pro-caspase-1, and of IL-1β and IL-18, co-localized with glia maturation factor (GMF), APOE-ε4, sequestosome 1 (SQSTM1)/p62, LC3-positive autophagic vesicles, and LAMP1, a lysosomal marker. Notably, clusters of GMF overexpressing reactive astrocytes surrounded the amyloid senile plaques. GMF is a highly conserved proinflammatory protein that activates glial cells advancing human neurodegenerative processes. Conversely, in AD-model animals, GMF suppression mitigated the neurodegeneration. Altogether, these results showed that in humans, GMF could intensify NLRP3-driven neuroinflammation while concurrently hampering the autophagosomal pathway clearing Aβs aggregates [349]. Of note, Ahmed et al. [372] and Ramaswamy et al. [352] posited that GMF may advance neuroinflammation in all neurodegenerative diseases.

By sharp contrast, the results of another human postmortem study negated NLRP3 inflammasome function in the brains of advanced AD cases in which astrocyte activation was instead prominent [132]. 

In addition to Aβs and neuroinflammation, p-Taues are among AD’s main drivers. Stancu et al. [373] and Ising et al. [71] proved that a causal link existed between p-Taues and inflammasomes’ activation. They showed that following microglial endocytosis and lysosomal sorting, prion-like Tau seeds activated NLRP3 inflammasome signaling in the THY-Tau22 transgenic mouse line, a tauopathy-model animal. Moreover, the chronic intraventricular administration of NLRP3 inhibitor MCC950 significantly thwarted the neuropathology driven by the exogenous p-Tau seeds. Concurrently, NLRP3 suppression decreased the p-Taues levels and hindered their aggregation into neurofibrillary tangles by restraining Tau kinases’ activity while increasing that of p-Tau phosphatases [71]. Then again, Jiang et al. [60] showed that p-Tau paired-helical filaments and p-Taues from human tauopathy brains primed and activated IL-1β production via MyD88 and NLRP3•ASC•caspase-1 pathways in primary human microglia. The authors also showed that p-Taues accumulation concurred with elevated ASC and IL-1β levels in postmortem brains of tauopathies patients. 

Autophagy is a conserved process by which lysosomes remove dysfunctional cellular components and relevantly regulate NLRP3’s role in inflammatory CNS diseases [10,374]. A reduced biogenesis and function of lysosomes/autophagosomes promotes the NLRP3’s inflammasome activation driving the neuroinflammatory response in AD-model animals and cultured neural cells. In keeping with this, Zhou et al. [375] showed that overexpressing the transcription factor EB (TFEB), the primary regulator of lysosomal biogenesis, both improved the autophagosomes/lysosomes function and mitigated the neuroinflammation in AD-model cells.

Summing up, NLRP3 inflammasome targeting might hinder AD’s etiopathogenetic tripod, i.e., Aβs, p-Taues, and neuroinflammation, and beneficially affect tauopathies too. This is indeed a sensible proposal, but hitherto its real effectiveness in stopping human AD’s progression is unproven. Moreover, it does not consider inflammasomes’ plurality, potential functional interchangeability, and their different expression levels in the distinct neural cell types.

### 3.2. Parkinson’s Disease (PD) 

PD is the second-most-common age-related human neurodegenerative disorder. The progressive spread of PD neuropathology causes motor disturbances and neuropsychiatric disorders (e.g., depression). PD’s hallmarks are inclusions rich in misfolded α-synuclein (α-Syn) protein localized at the presynaptic terminals of melanin-rich dopaminergic neurons within the mesencephalic substantia nigra and subcortical corpus striatum. Zhang et al. [376] found the overexpression of IL-1β and IL-18 in cerebrospinal fluid samples from PD patients. Consistently, α-Syn mediated NLRP3 inflammasome activation in cultured human microglia [64]. In PD-model animals, β-hydroxybutyrate, a ketone body, did not inhibit NLRP3 [377] while blocking it in AD [378]. Therefore, α-Syn aggregates trigger chronic neuroinflammation sustained by mitochondrial dysfunction causing ROS overproduction and by unrestrained microglia activation advancing dopaminergic neurons’ pyroptosis [379,380,381].

**Figure 3:**
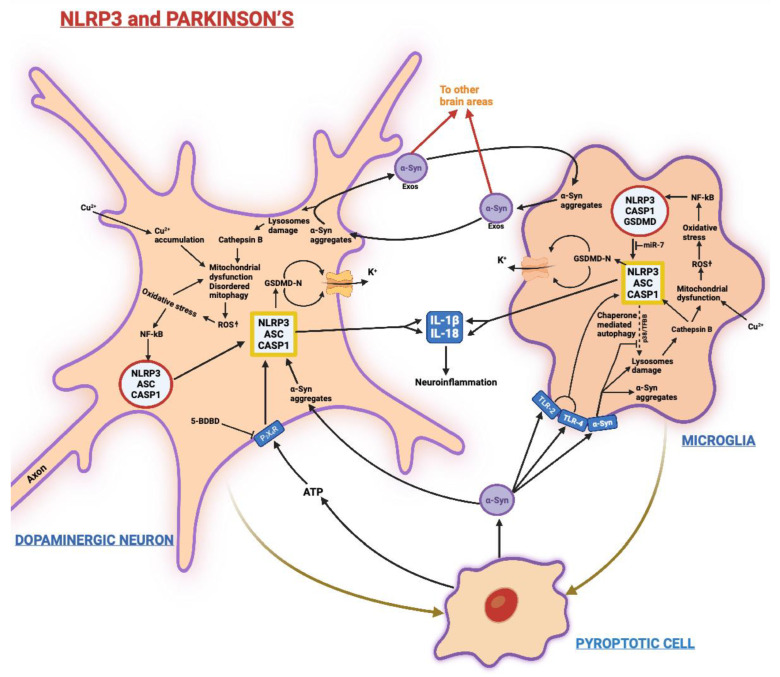
Summary illustration of stressors and factors inducing/modulating dopaminergic neurons’ and microglia’s NLRP3 inflammasome activation and its consequences in PD. Overproduced α-synuclein (α-Syn) forms cytosolic aggregates (named when massive Lewis bodies) that damage lysosomes releasing cathepsin B, a cysteine protease. The latter interferes with mitochondrial activities causing in sequence dysfunctional mitophagy, ROS surpluses, oxidative stress, NF-κB pathway signaling, and overexpression of NLRP3 inflammasome components, the latter’s activation, and its downstream consequences. Exogenous ATP from pyroptotic cells helps activate NLRP3 inflammasome via the P2X_7_ purinergic receptor signaling (see Box 2 for more details). The upshots are the release of IL-1β and IL-18 and K^+^ efflux through pores made of GSDMD-N terminal fragments. α-Syn is also released extracellularly within exosomes that spread and are taken up by neighboring neural cells, expanding the neuropathology, or they circulate in the body fluids thus affecting peripheral tissues. Accumulated Cu^2+^ ions also harm mitochondria contributing to NLRP3 inflammasome’s activation. The toxic α-Syn effects are similar in microglia, in which they are mediated by TLR-2 and TLR-4 receptors too. α-Syn also blocks the chaperone-mediated autophagy (CMA) pathway regulated by the p38 MAPK/TEFB axis. Eventually, both nigrostriatal dopaminergic neurons and microglia undergo pyroptotic death. A yellow frame encloses the assembled inflammasomes, while nuclear envelopes are orange-colored. Abbreviations: ASC = apoptosis-associated speck-like protein endowed with a CARD domain; 5-BDBD = 5-(3-Bromophenyl)-1,3-dihydro-2H-benzofuro[3,2-e]-1,4-diazepin-2-one; CASP1 = caspase-1; Exos = exosomes; GSDMD-N = gasdermin D N-terminal fragments; NF-κB = nuclear factor κB; P2X_7_ = purinergic receptor; p38 MAPK = p38 mitogen activated protein kinase; ROS = reactive oxygen species; TFEB = transcription factor EB; TLR=Toll-like receptor. ↑ROS = increase in ROS levels. ⊥ = inhibition. The other arrows show the sequences of molecular events induced by stressors and factors.

Figure 3 sums up the main signaling pathways involving NLRP3 in PD.

Moreover, Scheiblich et al. [382] reported that the signaling triggered by the binding of α-Syn monomers or, to a lesser extent, α-Syn oligomers to TLR-2 and TLR-5 receptors activated the NLRP3 inflammasome in microglia with no priming needed. Using immunohistochemical and genetic approaches, von Herrmann et al. [383] supplied evidence that dopaminergic neurons are sites of NLRP3 activity in PD. Moreover, increases in NLRP3 inflammasome and NLRP3-dependent pro-inflammatory cytokines were detectable in the peripheral plasma of PD patients, proving NLRP3 inflammasome involvement in PD’s pathogenesis [196,384]. The latter authors also showed that miR-7 inhibited NLRP3 gene expression in microglia, thereby reducing microglia activation, neuroinflammation, and nigrostriatal dopaminergic neuron pyroptosis. A patient-based study characterized NLRP3 in the first stages of midbrain nigral neurodegeneration and in the biofluids drawn from PD patients, suggesting that NLRP3 may be both a key inflammation mediator in the degenerating midbrain and a tractable therapeutic target [385]. Moreover, Wang et al. [386] showed that NLRP3 activation and IL-1β and IL-18 maturation occurred in the 6-OHDA (6-hydroxydopamine) neurotoxin-induced PD-model rat. The purinergic P2X_4_-R siRNA-knockdown or block by the specific antagonist 5-BDBD (5-(3-bromophenyl)-1,3-dihydro-2H-benzofuro[3,2-e]-1,4-diazepin-2-one) counteracted NLRP3’s effects, alleviated neuroinflammation, and reduced dopaminergic neuron pyroptosis. Therefore, the authors posited that the ATP•P2X_4_-R signaling drives NLRP3 inflammasome’s activation, which next regulates glial cell activation, nigrostriatal dopaminergic neurodegeneration, and dopamine levels (Figure 3; see also more details and the literature in Box 2) However, here one should be wary of extrapolating these data to PD patients. In PD-model rat brains, NLRP3 inflammasome’s activation is not in fact equivalent to that proper of human PD brains. The present understanding of any beneficial effects of antagonizing ATP•P2X_4_-R’s signaling is too limited. Therefore, we need more studies to assess the pathophysiological relevance of nigrostriatal ATP•P2X_4_R signaling in humans.

Consistently, inhibiting NLRP3 function with MCC950 evoked substantial neuroprotection in the 6-OHDA PD-model rats [387] and in MPTP (1-methyl-4-phenyl-1,2,3,6-tetrahydropyridine)-induced PD-model mice [384]. Moreover, NLRP3 inflammasome’s activation in microglia promoted the extracellular release of α-Syn-conveying Exos, which could advance α-Syn spreading in PD brains [388]. Interestingly, copper (Cu^2+^) accumulation also advanced PD’s pathogenic mechanisms by inducing ROS-mediated oxidative stress, activating the NF-κB-p65 pathway in BV2 microglial cells [49]. A persistent intracellular Cu^2+^ buildup upregulated the NLRP3 pathway-related proteins, advancing proinflammatory cytokine secretion and a disordered mitochondrial autophagy (or mitophagy), altogether resulting in dopaminergic neuron pyroptosis. Of note, Cu^2+^ may drive AD neuropathology as well [48].

Finally, despite extensive investigations into the NLRP3 inflammasome-activating mechanisms in the diverse inflammatory brain diseases, their regulatory networks are still unclear in microglia and other neural cell types. Chen et al. [389] showed that NLRP3 is a substrate of chaperone-mediated autophagy (CMA). The p38/TFEB (transcription factor EB) axis regulated NLRP3 inflammasome degradation via CMA, inhibiting the overproduction of proinflammatory cytokines in microglial cells. Furthermore, both p38 and NLRP3 inhibitors could mitigate α-Syn aggregate-induced microglia activation and nigrostriatal dopaminergic neuron pyroptosis. Moreover, Panicker et al. [390] showed that the functional loss of Parkin, an E3 ubiquitin ligase, resulted in the priming and spontaneous activation of the NLRP3 inflammasome in mouse and human dopaminergic neurons, leading to their pyroptosis.

From a clinical standpoint, human PD is quite complex. Therefore, one may conclude that the roles of NLRP3 and other-than-NLRP3 inflammasomes in human PD require further investigations to be fully clarified and integrated to lead to effective therapeutic interventions.

### 3.3. Multiple Sclerosis (MS) and Experimental Autoimmune (or Allergic) Encephalomyelitis (EAE)

MS is a chronic autoimmune disease of unclear etiology affecting both the brain and spinal cord whose hallmarks include focal (plaque) demyelination and chronic neuroinflammation/neurodegeneration. One accredited theory posits that patients’ T cells attack myelin sheath antigens, causing MS. The suggested relationship between MS and the NLRP3 inflammasome has linked autoimmunity with innate immunity and neuroinflammation [391,392,393,394,395]. Moreover, as gain-of-function genetic variants of the NLRP3 (e.g., Q705K) and NLRC4 inflammasomes associate with a more severe MS course, a constitutive NLRP3 inflammasome activation could be a risk factor for clinical MS presentation [396]. Moreover, Vidmar et al. [397] highlighted as pathogenetically important for MS patients the increased burden of rare variants in (i) *NLRP1* and *NLRP3* genes; (ii) genes partaking in inflammasome downregulation via autophagy and IFN-β; and (iii) genes involved in responses to type-1 IFNs (e.g., *PTPRC*, *TYK2*) and to DNA virus infections (e.g., *DHX58*, *POLR3A*, *IFIH1*).

Keane et al. [398] and Voet et al. [215] showed that following NLRP3 inflammasome activation, there occurred an increased IL-1β gene expression within MS demyelination plaques coupled with elevated levels of ASC, caspase-1, and IL-18 in the brains and cerebrospinal fluids of MS patients. Moreover, NLRP3 inflammasome pathway-related components were overexpressed in the blood monocytes isolated from the minor fraction of patients suffering from primary progressive (i.e., with no alternation of pauses and relapses) MS (PPMS), so entailing increased IL-1β production [393,394,399]. These results showed IL-1β as a prognostic factor in PPMS patients and the NLRP3 inflammasome as a prospective therapeutic target. Thus, a specific NLRP3 inhibitor may improve MS histopathology and reduce myelin sheath damage.

According to Farooqi et al. [400], EAE is a proper mouse model for pathogenetic and pharmacotherapeutic studies into human MS molecular mechanisms. In EAE-model mice, NLRP3 inflammasome’s activation critically induced T-helper cell migration into the CNS. Next, the activated NLRP3 inflammasome of primed T cells (and microglia) drove the release of proinflammatory cytokines, thus partaking in MS pathogenesis [394,401]. In EAE-model mice the NLRP3 inhibitor MCC950 prevented the conversion of CNS astrocytes to the A1 neurotoxic reactive phenotype otherwise induced via the NF-κB pathway-mediated IL-18 production. Consistently, after the systemic delivery of NLRP3 inhibitor MCC950 axonal injury was mitigated within lysolecithin-induced demyelinated lesions in mice [402,403]. MCC950 also hindered complement C3 protein release from the astrocytes, which would have otherwise impaired hippocampal neuron viability [404]. IFN-β administration did improve this NLRP3-dependent EAE form. Conversely, when ad hoc experimental regimens brought about a NLRP3-independent, more aggressive EAE, the IFN-β treatment was ineffective. A similar NLRP3-independent mechanism might be at work in human MS cases not profiting from IFN-β therapy [405].

In conclusion, there is an intensely felt need to expand the study of NLRP3 and other-than-NLRP3 inflammasomes’ role(s) in MS, using human neural cell-based experimental models to achieve a more detailed molecular picture and identify disease-modifying therapeutic targets.

### 3.4. Amyotrophic Lateral Sclerosis (ALS) 

ALS is a devastatingly progressive multifactorial disorder characterized by the primary degeneration of the cerebral motor cortex, brain stem, and spinal cord motoneurons leading to skeletal muscle atrophy and paralysis. ALS patients may also develop cognitive and behavioral changes due to neurodegeneration-affected subcortical areas, e.g., diencephalon’s dorsal thalamus. Typically, 90% of cases occur sporadically, and their etiological factors are poorly defined (smoking, violent sports, military service, exposure to insecticides and pesticides). About 10% of ALS cases are familiar due to heritable mutated genes. *SOD1* (superoxide dismutase 1) gene mutations occur in 20% of familiar cases [406]. The current belief is that *SOD1* mutations only trigger ALS onset within motoneurons but elicit only delayed and minor harm [407]. However, in astrocytes and/or microglia, *SOD1* mutations advance ALS progression [408]. TDP-43 (transactive response DNA binding 43 kDa) protein could be another ALS etiological agent as it accumulates in both sporadic and familial cases [409]. TDP-43 forms toxic ubiquitinated aggregates in the cytoplasm of neural cells of both ALS and frontotemporal lobar degeneration (FTLD) patients [410,411]. Neurons and astrocytes can secrete mutated or oxidized SOD1 and TDP-43 as misfolded proteins, which activate microglia by interacting with CD14, TLR-2, TLR-4, and scavenger receptors [412,413]. Thus, exogenous whole or fragmented, wild-type or mutated TDP-43 bound microglia’s CD14 cell surface receptor activating AP1 and NF-κB pathways and upregulating NOX2 (SOD-generating NADPH oxidase 2), TNF-α, NLRP3•ASC•caspase-1, and IL-1β release. Importantly, TDP-43 was toxic to motoneurons only in the presence of microglia presence [414]. Using in situ hybridization and immunocytochemistry, Banerjee et al. [415] showed that an upregulated NLRP3 inflammasome occurred in neurons and glia of cognitively impaired ALS patients. Conversely, no differences were detectable between cognitively resilient ALS and healthy subjects.

**Figure 4:**
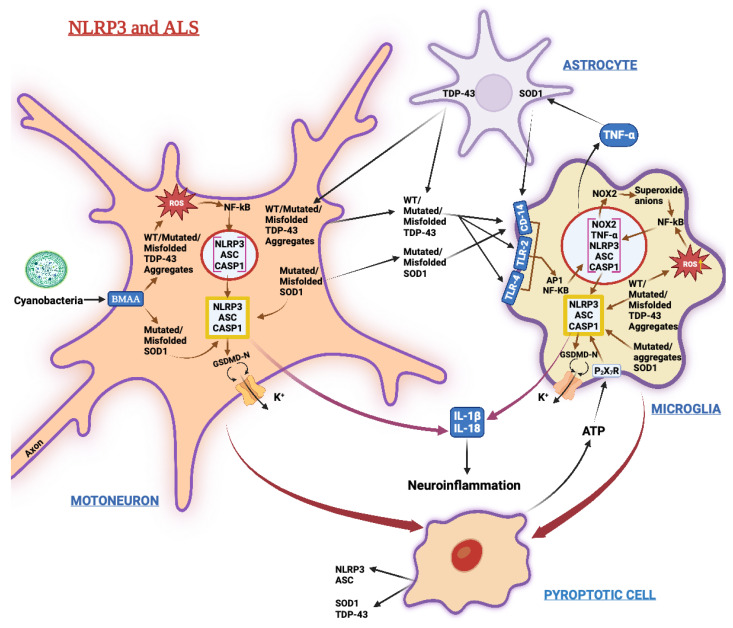
Summary depiction of stressors and factors inducing/modulating motoneurons’ and microglia’s NLRP3 inflammasome’s activation and its consequences in ALS. Mutated/misfolded SOD1 and TDP-43 proteins as variously sized aggregates damage mitochondria, causing in sequence ROS surpluses release, oxidative stress, and NF-κB pathway signaling. These lead to NLRP3 inflammasome’s component overexpression, NLRP3 inflammasome activation, over-release of IL-1β, IL-18, K^+^ efflux, and eventually motoneurons’ and microglia’s pyroptosis. Exposure to the toxic BMMA amino-acid released by *Cyanobacteria* worsens the toxic effects of misfolded/mutated SOD1 and TDP-43. Toll-like receptors and CD-14 bind misfolded/mutated SOD1 and TDP-43 activating the AP1/NF-κB axis, and the expression and activation of NLRP3 inflammasome’s components. ATP from pyroptotic cells partakes in NLRP3 activation via P2X_7_ receptor signaling (see Box 2 for details). Astrocytes also release misfolded/mutated SOD1 and TDP3 that are engulfed by other neural cells, thus spreading the neuropathology. Besides ATP, pyroptotic cells also release NLRP3, SOD1, TDP-43, and ASC proteins that contribute to the neuroinflammation. A yellow frame encloses the assembled inflammasomes, while nuclear envelopes are orange-colored. Abbreviations: AP1 = activator protein 1; ASC = apoptosis-associated speck-like protein endowed with a CARD domain; BMAA = β-methylamino-L-alanine; CASP1 = caspase-1; CD14 = cluster of differentiation 14; GSDMD-N = gasdermin D N-terminal fragments; NF-κB = nuclear factor κB; NOX2 = NADPH oxidase 2; P2X_7_R = purinergic receptor; ROS = reactive oxygen species; SOD1 = superoxide dismutase 1; TDP-43 = TAR DNA-binding protein 43; TLR− =Toll-like receptor−; TNF-α = tumor necrosis factor-α; WT = wild-type. The arrows show the sequences of molecular events induced by stressors and factors.

Figure 4 sums up the main signaling pathways involving NLRP3 in ALS.

Johann et al. [127] showed that an activated NLRP3 inflammasome concurred with elevated levels of caspase-1, IL-1β, and IL-18, particularly in the spinal cord astrocytes of the SOD1G93A ALS-model mice and in the serum and spinal cord tissue of sporadic ALS patients—altogether findings confirming NLRP3 inflammasome’s involvement in ALS. Moreover, Kadhim et al. [416] found that IL-18 was upregulated in the cerebral tissue of sporadic ALS patients vs. age-matched controls. Furthermore, Gugliandolo et al. [417] strengthened the concept that neuroinflammation plays a crucial role in ALS by confirming NLRP3 inflammasome activation and its sequels in SOD1G93A ALS-model rats. Immunofluorescent studies conducted on symptomatic SOD1G93A ALS-model mice revealed that NLRP3 and ASC expression intensity increased along with ALS progression, proving NLRP3’s involvement in neuron death [418]. Moreover, Michaelson et al. [419] suggested a novel ALS pathogenetic mechanism mediated by the amino acid β-N-methylamino-l-alanine (BMAA), a *Cyanobacteria* product. BMAA is not a protein constituent, but a powerful neurotoxin inducing protein misfolding, NLRP3 inflammasome activation, and proinflammatory cytokine overexpression in spinal motoneurons.

In their work, Van Schoor et al. [420] observed increases in the NLRP3 inflammasome, GSDMD-N fragments, and IL-18 in the motor cortex and spinal cord microglia of human ALS patients, which suggested that an activated NLRP3 inflammasome had triggered the cells’ pyroptosis. As compared to controls, in human ALS samples, a reduced array of neurons matched with an increased throng of cleaved-GSDMD-positive microglial cells in the underlying white matter of the premotor cortex. No alike findings were obtained in the human spinal cord. Similar findings were made in the cortex of TDP-43A315T transgenic mice in model ALS and FTLD [421]. In addition, these results stressed the relevance of ROS and ATP generation, both potential therapeutic targets, for microglial NLRP3 inflammasome activation and neuronal pyroptosis, which was confirmed in SOD1G93A-induced ALS-model mice. Importantly, both wild-type and mutant TDP-43 proteins activated the overexpressed NLRP3 and its downstream effects in the microglia of SOD1G93A mice. This proved that NLRP3 is the crucial microglial inflammasome mediating SOD1G93A-induced pyroptosis [65].

Lacking a suitable human microglia model, Quek et al. [422] characterized peripheral blood monocyte-derived microglia-like cells (ALS-MDMi) isolated from ALS patients at various stages. Importantly, ALS-MDMi recapitulated ALS neuropathology hallmarks, i.e., abnormal phosphorylated and non-phosphorylated TDP-43 cytoplasmic accumulation and phagocytosis impairment that paralleled ALS progression; altered neuroinflammatory cytology; DNA damage; NLRP3 inflammasome’s activation; and microglia pyroptosis.

It is seemly to consider the studies about NLRP3 and other-than-NLRP3 inflammasomes in human ALS are still in a preliminary phase even in the light of the groundbreaking results reported by Van Schoor et al. [420]. The latter should encourage scientists to delve deeper into the pathogenetic mechanisms of this devastating disease to find novel effective therapeutic approaches.

### 3.5. Huntington’s Disease (HD)

HD is a rare autosomal dominant neurodegenerative disease caused by the unstable CAG repeat expansion in the Huntington (*HTT/IT15*) gene and presenting with motor, cognitive, and psychiatric symptoms [423] When the *HTT/IT15* gene holds 39 to 180 CAG repeats, the translated polyglutamine-containing mutant HTT protein (mHTT) complexes with and disrupts the normal function of several transcription factors, thereby altering the activities of neurons, astrocytes, and microglia. HD’s harming mechanisms include mitochondrial dysfunction, excitotoxicity, CREB and BDNF downregulation, and microglia activation, altogether advancing neuronal death by apoptosis, necroptosis, ferroptosis, and NLRP3-linked pyroptosis [424,425].

Various HD-model animals were set up to clarify its molecular mechanisms and to try novel therapeutics for it. The transgenic R6/2 (B6CBA-Tg[HDexon1]62Gpb/1J) mouse line expressing the human *HTT* gene exon 1 carrying 120 ± 5 CAG repeats is the most popular HD animal model [426]. An upregulated NLRP3 inflammasome and caspase-1 expression already occurred in 13-week-old R6/2 HD-model mice, particularly in striatal parvalbumin interneurons and spiny GABAergic neurons, which preferentially undergo pyroptosis in HD [427]. Poly(ADP-ribose) polymerase-1 (PARP-1) is a nuclear enzyme whose activity is crucial for DNA repair in humans. Olaparib, a PARP-1 inhibitor presently sold as an anti-tumor drug, could also regulate NLRP3 inflammasome activation in the R6/2 HD-model mice. When given from the pre-symptomatic stage onwards, Olaparib mitigated neuronal pyroptosis, neurological symptoms, and neurobehavioral tests results, lengthening the survival of HD-model mice. Therefore, Olaparib could help human HD too [428]. Moreover, Chen et al. [429] showed that NLRP3 inhibitor MCC950 given to R6/2 HD-model mice suppressed IL-1β and ROS overproduction, mitigating neuroinflammation, motor dysfunction, and neuronal pyroptosis, while upregulating PSD-95 and NeuN proteins, and lengthening animals’ lifespans. Therefore, inhibition of NLRP3’s signaling, and its downstream effects would be therapeutically helpful in HD.

Interestingly, a role in HD etiopathogenesis may be played by galectins (i.e., “*S-type lectins*”)—soluble proteins specifically binding β-galactoside carbohydrates and playing multiple roles in autophagy, immune responses, and inflammation. Siew et al. [430] reported that galectin-3 (Gal-3) plasma levels increased well over healthy controls in HD patients and HD-model mice. In HD-mice, microglia Gal-3 levels increased prior to motor symptom presentation and stayed high while HD progressed. Gal-3 co-localized with microglial lysosomes, blocked the autophagic elimination of damaged endolysosomes, and partook in neuroinflammation via the NF-κB/NLRP3 axis. Gal-3 knockout improved HD-related neuropathology and survival in HD-model mice, showing Gal-3 as a potential therapeutic target. Conversely, Gal-1 and Gal-8 hindered neuroinflammation, promoting neuroprotective effects [431].

HD’s rare occurrence is an adjunct hurdle to studies about the roles played in it by NLRP3 and other inflammasomes. However, this circumstance should not discourage attempts to increase our insights in this ailment, both in patients and animal models.

## 4. Brain NLRP3 and Neurotropic Viruses Infections

Both DNA and RNA neurotropic viruses activate the brain’s NLRP3 inflammasome, causing neuroinflammation and sometimes triggering chronic neurodegenerative diseases [75]. Here, we review a few neurotropic viruses playing NLRP3-linked roles in human neuropathology.

### 4.1. Zika Virus (ZIKV) Encephalitis

The Zika Virus (ZIKV) is a single-stranded positive-sense RNA arbovirus of the *Flaviviridae* family (*Flavivirus* genus that also includes Dengue, West Nile, Yellow Fever, and Japanese Encephalitis viruses). ZIKV associates with congenital microcephaly in newborns and Guillain–Barré syndrome, myelopathy, and encephalitis in adults. Tricarico et al. [432] showed that ZIKV infected the U87-MG glioma cell line causing NLRP3 inflammasome activation and IL-1β oversecretion. Consistently, He et al. [82] made the same observations in the brains and sera of ZIKV-infected mice. ZIKV’s NS5 protein drove ROS overproduction and NLRP3 inflammasome assembly, both needed for its activation. Conversely, in vitro and in vivo NLRP3 deficiency upregulated type-I IFN and strengthened the host’s resistance to ZIKV, confirming NLRP3’s role in ZIKV infection [433,434].

### 4.2. West Nile Virus (WNV) Encephalitis

Another *Flavivirus*, the West Nile Virus (WNV), causes an encephalitis entailing neurons’ death and elevated IL-1β plasma levels. In a mouse model, WNV infection briskly induced IL-1β synthesis in cortical neurons. However, by cooperating with type-I IFN, the intensified IL-1β•IL-1β-R (receptor) signaling suppressed neuronal WNV replication, reducing the WNV brain load. Therefore, the NLRP3/IL-1β•IL-1β-R pathway regulated neuronal WNV infection and revealed a novel IL-1β antiviral action [435].

### 4.3. Japanese Encephalitis Virus (JEV)

By breaking the BBB, the Japanese Encephalitis Virus (JEV) enters the CNS where it induces a diffuse neuroinflammation. Thus, JEV infection activated (i) a ROS-dependent Src/Ras/Raf/ERK/NF-κB signaling axis in neurons/glia co-cultures [81]; (ii) a ROS/Src/PDGFR/PI3K/Akt/MAPK/AP-1 axis [436] and a PAK4/MAPK/NF-κB/AP-1 axis [437] in rat brain astrocytes; and (iii) via TLR-3 and RIG-I the ERK/MAPKp38/AP-1/NF-κB axis, ROS overproduction, and K^+^ efflux in cultured mouse microglia. These effects both triggered NLRP3 inflammasome signaling and polarized microglia toward the proinflammatory/neurotoxic M1 phenotype. In all instances, JEV advanced cytokine overproduction and neural cell pyroptosis [438].

### 4.4. Human Immunodeficiency Virus-1 (HIV-1) Encephalitis

The immunosuppressive Lentiviruses efficiently infect macrophages and lymphoid cells. Human Immunodeficiency Virus-1 (HIV-1) belongs to the Retroviridae family (*Lentivirus* genus). Burdo et al. [439] showed that during the primary infection, HIV-1 productively infects brain macrophages and microglia. Studies using primary human microglia showed that IL-1β was released after HIV-1 infection. Walsh et al. [440] proved that HIV-1 infection induced an NLRP3 inflammasome-dependent ASC translocation, caspase-1 activation, and mature IL-1β release from cultured microglia. The authors highlighted the need to analyze the inflammasome inhibitors’ effectiveness as novel therapeutics for HIV-1/AIDS.

### 4.5. Viroporin Proteins

Various RNA viruses, including *Coronaviridae*, express the viral-replication-indispensable small viroporin proteins. Being liposoluble, viroporins assemble hydrophilic transmembrane pores, allowing ions and/or small solutes to bidirectionally migrate along their electrochemical gradients. Viroporin activity could act as the “second signal” by increasing [Ca^2+^]_i_  or lowering the cytosolic pH due to H^+^-releasing ion channel activity in the lysosomal acidic compartment [441].

### 4.6. Encephalomyocarditis Virus (EMCV)

The Encephalomyocarditis Virus (EMCV) of the *Cardiovirus* genus (Picornaviridae family) is a non-enveloped, positive single-stranded RNA virus. Via an unclear sensing mechanism, the NLRP3 inflammasome detects EMCVs [442,443]. In this regard, Ito et al. [89] reported that by releasing Ca^2+^ from intracellular stores into the cytosol, ECMV’s viroporin ORF2b (or open reading frame 2b) triggered NLRP3 inflammasome activation.

### 4.7. SARS-CoV-2 Encephalitis

SARS-CoV-2 belongs to the β-*Coronavirus* genus (Coronaviridae family, also including 2003 SARS-CoV and 2012 MERS (Middle East Respiratory Syndrome)-CoV). SARS-CoV-2 is an enveloped single-stranded positive-sense RNA virus causing the COVID-19 (Coronavirus Disease 2019). The virus infects a wide spectrum of cell types. In the presence of Ca^2+^, SARS-CoV-2’s spike S1 glycoprotein binds ACE2 (angiotensin converting enzyme 2) and CD147 (cluster of differentiation 147) proteins, promoting virus endocytosis. Moreover, SARS-CoV-2’s envelope (E) protein binds TLR-2, which also helps promote AD and PD [444]. Earlier epidemics proved Coronaviruses’ neuroinvasive capability in humans [445,446]. SARS-CoV-2 infects neurons, astrocytes, microglia, and the BBB’s endothelial cells [447,448]. Notably, microglia and astrocytes are major sources of proinflammatory cytokines. Moreover, Sepehrinezhad et al. [449] found SARS-CoV-2 virions in the cerebrospinal fluid of COVID-19 patients presenting severe neurological symptoms previously affected or unaffected by neuropathologies and in “long COVID” patients [450]. However, in the healthy CNS, ACE2 expression is weak, prevailing in the brainstem’s respiratory centers—which explains the high prevalence of respiratory distress in COVID-19 patients [451]. However, uninfected AD patients showed upregulated ACE2 expression in the temporal and occipital neocortex and hippocampal CA1 subfield archicortex [452]. This ACE2 overexpression could advance SARS-CoV-2 infection in the same AD-hit inflamed areas, thus contributing to the high COVID19 mortality rates in aged AD patients [451].

Hitherto, SARS-CoV-2’s priming triggers are uncertain. Theobald et al. [453] showed that S1 spike glycoprotein initiated NLRP3 inflammasome activation. Other SARS-CoV-2 proteins—i.e., S, N, E, and the pore-forming viroporins ORF3a and ORF8—are also NLRP3 activators by causing K^+^ efflux and mitochondrial ROS over-release [83,84,85,86,87,88]. Moreover, Xu et al. [454] proved that viroporin ORF3a primed and activated the NLRP3 inflammasome through both ASC-dependent (canonical) and ASC-independent (noncanonical) pathways.

Notably, COVID-19 infection triggers a severe innate immune response producing elevated levels of multiple cytokines (“cytokines storm”) and inflammatory mediators (e.g., IL-1β, IL-2, IL-2-R, IL-4, IL-10, IL-18, IFN-γ, C-reactive protein, GCSF (granulocyte colony-stimulating factor), IP10, MCP-1, MIP-1α, and TNF-α). BV2 microglial cells exposed to SARS-CoV-2’s S1 spike glycoprotein expressed elevated levels of IL-1β, TNF-α, IL-6, NO, NLRP3, NF-κB signaling, and caspase-1 activity [88,455]. These cytokines cross the BBB inducing leukocyte infiltration, mitochondrial dysfunction, neuroinflammation, and neurons’ pyroptosis [442]. Interestingly, a mix of melatonin, vitamin C, and Zn^2+^ inhibited SARS-CoV-2-driven inflammasome activation, hindering the cytokine storm in animals [456].

Additionally, Ding et al. [457] proved that hypercapnia enhanced NLRP3 inflammasome activation and IL-1β expression only in hypoxic BV-2 microglia cells. Therefore, the hypercapnia resulting from lung-protective ventilatory strategies used in acute respiratory distress syndrome (ARDS) patients may lead to neuroinflammation and cognitive impairment via a microglial NLRP3/IL-1β-dependent mechanism.

Based upon the above findings, Heneka et al. [458] posited that NLRP3 inflammasome activation during COVID-19 heightens the risk for the later development of chronic neurodegenerative diseases. Independent clinical and epidemiological investigations indicated that SARS-CoV-2 infection and the ensuing “long COVID” tightly relate to the onset of AD, PD, prion disease (PrD), and other ailments, particularly in patients in advanced age or suffering from intercurrent illnesses (CVD, T2DM, hypertension, other neurological disorders) or severe/fatal COVID-19 [459,460,461]. Even more alarming, the receptor-binding domain of SARS-CoV-2’s S1 spike glycoprotein presents prion-like sequences. The latter diverge among viral variants, show a different affinity for ACE2, and promote immune-evasion, protein clustering, and protein aggregates’ “seeding”. The upshots would include prion-like proteins spreading, progressive dementia, or fast-evolving CJD [462,463,464].

Obviously, here we have considered only some of the known neurotropic viruses. The field of human brain-infecting viruses is more variegated and might also further expand in the future. Our knowledge about viral neuropathology is, we must admit, limited, particularly because viruses can target all stages of human life, from the uterus onward, with different age-related upshots. There is also a field that for the sake of brevity we omitted considering, i.e., the interactive relations between oncogenic viruses and inflammasomes, which deserves attention because of its potentially significant reflections on therapeutic outcomes.

## 5. Comments and Future Perspectives

An old dictum states that every disease starts with an inflammation. The prevalence of neuroinflammatory disease has been epidemically rising because of a lengthened lifespan and of little-appreciated toxic, environmental, and lifestyle-linked factors. To worsen this bleak situation, acute brain illnesses (e.g., stroke, hemorrhage, infection) too can trigger chronic neuroinflammation/neurodegeneration in a significant fraction of patients [465]. A steadily growing literature attests that NLRP3 inflammasome activation in CNS microglia and circulating monocytes plays a pivotal role in promoting the neuroinflammation driven by a host of etiologic factors (q.v. Table 1), potentially advancing the progression of neurodegenerative diseases [27,466,467]. Conversely, NLRP3’s roles in the other neural cell types (i.e., neurons, astrocytes, and oligodendrocytes) [3,468,469,470] and in CNS pericytes and endothelial cells [126,471] have received less attention, probably because such cells preferentially express other types of inflammasomes. In fact, NLRP3 activity in such cells is modest and/or is the object of controversy, particularly in astrocytes, although NLRP3’s inhibition still gives some therapeutic advantage. Moreover, these same neural cell types more intensely express various other-than-NLRP3 inflammasomes. The latter can also exert significant neuroinflammation-sustaining effects, as specific NLRP3 inhibitors do not hinder other-than-NLRP3 inflammasomes’ activities [24]. We previously reviewed the known roles of various other-than-NLRP3 inflammasomes in human brain disease [24]. That work inspired us to delve deeply also into the role(s) of the brain’s NLRP3 inflammasome. Indeed, the NLRP3-related extensive research works herein reviewed shows the high complexity of both the regulatory mechanisms involved and of the physiological, pathological, and ethnic/pharmacological factors that promote or hinder its activation. Particularly the abundance of blocking or preventative factors, many of them identified over millennia by TCM, bodes well for future therapeutic modulations of NLRP3 activity in various pathological settings. Various reports showed that particularly inhibiting microglial NLRP3 function exerted beneficial effects in rodent experimental models of human neurodegenerative illnesses. These favorable outcomes inspired and still inspire the opinion that therapeutically targeting the NLRP3 inflammasome will mitigate or stop both acute and progressive human neuroinflammatory diseases [472,473,474]. As just mentioned, despite or thanks to the intricacies of NLRP3 inflammasome’s activating mechanisms, there are plenty of agents modulating its activity (Table 2, Table 3 and Table 4). At present, many small molecules are undergoing pharmaceutical research/development as novel candidate drugs targeting the NLRP3 inflammasome in various diseases [274]. At least five companies have started ad hoc clinical trials, of which Inflazome and NodThera have reported Phase I positive results of their brain-penetrating NLRP3 inflammasome inhibitors (Inzomelid [251] and NT-0796 [274], respectively), expecting to use them to treat central and peripheral nervous inflammatory diseases. These discoveries have even raised the possibility of a common cure for all or at least some human brain diseases. Moreover, Lupfer and Kanneganti [21] reported the existence of inflammasomes, such as NLRC3, NLRP6, NLRP12, and NLRX1, which hinder NF-κB pathway activation, thereby mitigating or switching off the incumbent or ongoing neuroinflammation. Such “*anti-inflammasomes*” deserve more consideration because in a hopefully not too far future, their pharmacological activation by proper means (yet to be established) could be a valuable therapeutic asset that will switch off neuroinflammation through physiological mechanisms.

Therefore, the intuitive conclusion is that reality is more intricate than it might appear at first sight. Furthermore, uncertainties and controversies about the etiological mechanisms driving human neurodegenerative diseases help confound the picture, as do other problems that we will briefly discuss below.

(*i*) *Are inflammasomes functionally interchangeable?* Hitherto the interplays that might occur between or among the distinct inflammasomes expressed by each human neural cell type remain mostly undefined. Yet, it is necessary to clarify them to better assess the therapeutic impact of NLRP3 inflammasome inhibitors. Denes et al.’s [336] study results in mice called for caution, as they showed that inflammasomes (e.g., AIM2) can functionally overtake a blocked NLRP3 (Figure 1). A (partial) solution to this problem might entail targeting the ASC protein, which would hinder the activation of all canonical inflammasomes instead of those of NLRP3s only [475]. The inflammasomes’ noncanonical activation problem will persist but might be a minor one.

(*ii*) *The species difference problem*. Significant genomic differences apart, not all organs of humans and mammals are morpho-functionally alike. Acceptable similarities exist with liver, kidneys, and lungs. Yet, considering the CNS, while the human cerebral cortex consists mostly of a non-olfactory six-layered *neocortex*, the widely used rodent models have a less developed, structurally simpler, and mostly olfactory cortex. Moreover, fundamental cytological divergences in size, shape, connections, and functions distinguish the diverse types of neural cells of the human cortex from their rodent counterparts [476]. Human brain’s molecular regulatory mechanisms, e.g., those involved in receptor signal transduction [133] and inflammasome regulation [24,27,477] (see also Box 1 and Box 2), also remarkably diverge from those of rodents. Moreover, human neurodegenerative diseases do not plague rodents in nature. Importantly, in rodent models of human neurodegenerative diseases, the astrocytes undergo an early death—which justifies the often-little attention paid to them—while neurons keep surviving. Conversely, human neurodegenerative diseases kill neurons first, while astrocytes survive and help advance the neuropathologies. Hence, a tight genomic, proteomic, and bio-pathological conformity between animal and human brains is lacking [478,479]. Although brilliant and highly praiseworthy, the manifold animal models of human neurodegenerative diseases in existence cannot surmount such inter-species differences [480]. A quite low animal-to-human translation rate of brain disease-targeting drugs has been persisting for decades, being ascribed to preclinical studies’ faults in “internal consistency” (e.g., design flaws, uncontrolled bias) and/or “external consistency (i.e., animal models pre-testing). As a long trail of clinical trial failures shows, it is difficult to safely predict the effectiveness in humans of drugs pre-tested with favorable results in transgenic animal models [481]. Procedures involving animal models were necessary when nothing or truly little was known about human brain diseases. Now we know much more, albeit not yet enough. Moreover, in recent decades, the legislative/bureaucratic requirements to evaluate novel drugs have become increasingly burdensome to hinder the use of inadequately tested therapeutics. This trend has become stronger after rare events in which properly approved drugs unexpectedly elicited adverse reactions in the patients [482]. Moreover, the repurposing for neurodegenerative diseases of drugs previously evaluated for other ailments in clinical trials is not so easy to do, which precludes the faster testing of potentially useful drugs [483]. Hence, it would be wise to introduce some procedural changes. Animal and/or in silico studies should still help preselect lead drugs. Next, preclinical human untransformed neural cell models in vitro would allow for the assessment of the latter [24,141,212,484] prior to any clinical trial assessment. On rare occasions, animal studies might even be skipped in favor of preclinical human model studies [24,141,212,484]. Human neural cells models will help clarify specific etiopathogenetic mechanisms while supplying safer predicting information about effective drug benefits in clinical settings.

(*iii*) *Symptomatic and/or etiologic therapies?* Hitherto, no causal “brain disease modifying” therapies are available for human neurodegenerative diseases. An exception may be the just reported promising effects of Lecanemab, a humanized IgG1 monoclonal antibody binding soluble Aβ protofibrils. After 18 months, Lecanemab reduced brain amyloidosis and slowed cognition decline in early-stage AD patients vs. the placebo-given group. However, Lecanemab also caused collateral brain swelling and/or hemorrhage in some patients, particularly in case of APOE-ε4 homozygotes or anticoagulant therapy [485]. Hence, while Lecanemab’s results confirm that Aβs play a key pathogenetic role in human AD, further studies will prove its etiologic or symptomatic value regarding Aβs/p-Taues’ overproduction and accumulation and inflammasomes’ activity.

## 6. Conclusions

In recent years, neuroinflammation has been attracting a lot of attention, particularly concerning one of its mediators, i.e., the NLRP3 inflammasome. In the present work, we systematically review the huge and still mounting evidence related to both NLRP3’s involvement in human and animal models of acute and chronic brain diseases, and its many functional activators and inhibitors so far known. Unquestionably, no field expert should disregard the NLRP3 inflammasome, as it is intensely expressed by microglia and circulating monocytes. However, here we wish to stress the indisputable fact that human and animal neural cells of all types, whose morphologies and functions significantly diverge, also express many other inflammasomes and various "*anti-inflammasomes*"—the latter being tasked with mitigating neuroinflammation. Moreover, the so-called primary drivers of the distinct brain diseases should also be taken into due account because they can simultaneously trigger neurotoxicity and neuroinflammation. Hence, a more comprehensive view of the underlying molecular mechanisms of each brain disease would be beneficial. Importantly, the yet available data on the several inflammasomes’ roles in *human* brain diseases are limited and controversial. Therefore, this is a field widely open to groundbreaking investigations. We are confident that choosing human untransformed neural cells as models for pathogenetic and pharmacological studies will advance our knowledge about each neuropathology and hasten the achievement of effective etiological therapies.

## Figures and Tables

**Table 1:** Main conditions and factors activating the brain NLRP3 inflammasome.

Condition/Factor	Mechanisms	References
**Vascular ailments** Stroke Intracerebral hemorrhage Hemorrhagic stroke	Mitochondrial disfunction after hypoxic ischemia/reperfusion (HI/R) Chronic hypoxia	[32,33,34,35,36,37,38,39,40]
**Seizures** Mesial lobe temporal epilepsy Soman or A255 (nerve agent) exposure	Acetyl- and butyryl-cholinesterase inhibition	[41,42]
**Metal accumulation** Manganese (Mn), Lead (Pb) Copper (Cu) Cadmium (Cd) Aluminium/alum	Metal-induced neurotoxicity ↑^§^ ROS & NF-κB-p65 pathway CaSR and GCP6RA signaling	[43,44,45,46,47,48,49,50,51] See also Box 1
**Mechanical stresses and strains** Skull trauma Optic nerve trauma Elevated intracranial pressure Glaucoma	Osteopontin NIMA-related kinase 7 (or NEK7) P2X_7_ receptor activation HMGB1/caspase-8 pathway	[52,53,54,55,56,57,58,59] See also Box 2
**Neurodegenerative diseases** Alzheimer’s disease (AD) Tauopathies Parkinson’s disease (PD) Amyotrophic lateral sclerosis (ALS) Huntington’s disease (HD) Prion disease (PrP^Sc^)	Aβs, autophagy block, NEK7 p-Taues paired helical filaments ER stress, ↑ ROS α-Synuclein aggregates Mutated *SOD1*, *TDP-43* Expanded CAG repeats in *HTT/OT15* gene Prion protein seeding	[60,61,62,63,64,65,66,67,68,69,70,71]
**Environmental pollution** PM2.5	Increased ROS production by microglia	[72,73]
**Infectious diseases** Sepsis (bacteria, fungi) West Nile Virus (WNV) HIV-1 Herpes Virus 1 Japanese Encephalitis Virus (JEV) Zika Virus (ZIKV) SARS-CoV-2 Encephalomyocarditis Virus (EMCV) Tuberculosis	Bacterial and fungal toxins Intensified IL-1β signaling Tat and gp120 proteins Gasdermin D-dependent pyroptosis ROS-dependent activation of Src/Ras/Raf/ERK/NF-κB signaling axis NS5 protein and ↑ ROS S1 spike glycoprotein, viroporin ORF3a/8 viroporin ORF2b Early secreted antigenic target protein of 6 kDa (ESAT-6)	[74,75,76,77,78,79,80,81,82,83,84,85,86,87,88,89,90]
**Metabolic disorders** Atherosclerosis Gout Obesity/high-fat diet Nonalcoholic hepatic steatosis Type-2 diabetes mellitus (T2DM)	Hypercholesterolemia Urates→NEK7 Glucocorticoids and fatty acids surpluses→TNFR and Toll-like receptors ROS, NO, hydroperoxides, scavenger receptors, mTOR	[91,92,93,94]
**Iatrogenic factors** Postoperative cognitive dysfunction Cyclophosphamide cystitis GdCl_3_, cinacalcet Glucocorticoids (elevated levels)	Drugs, infection, electrolyte imbalance TNF-α Calcimimetic•CaSR/ERK1/2/CaMKII NLRP1 and NLRP3 inflammasomes	[95,96,97,98,99,100,101] See also Box 1
**Psychotropic drugs** Cocaine Methamphetamine Scopolamine Ethanol Morphine Fentanyl	*σ*-1 receptor TLR-4 ↑ *Dhx58*, *S100a*, *Lrm4* genes TLR-4 *μ*-3 and *κ* opioid receptors *μ*-opioid receptor	[102,103,104,105,106,107,108]
**Cellular stress and injury** ATP Pore-inducing agents Phagocytosed protein polymers ROS Cardiolipin Raised IL-1β levels Reduced cyclic AMP (cAMP) levels Zn^2+^ deficiency K^+^ efflux Ca^2+^ and Cl^−^ influx	Purinergic receptor signaling DDX3X protein/NLRP3 complexes Heat shock protein 60 (HSP60) and TLR-4-p38 MAPKs axis Oxidized mtDNA and proteins Lysosome-released cathepsin B Mitochondria-released hexokinase, ROS NLRP3 activation Ionic imbalances	[25,109,110,111,112,113,114,115,116,117,118,119]
**Aging** Inflammaging	↑ Membrane attack complexes (MAC) Reduced mitochondrial fission and fusion Declined mitophagy Mitochondrial damage Selective autophagy-mediated mitochondrial homeostasis (in microglia)	[33,112,120,121,122]

^§^ ↑ = increased.

**Table 2:** RNAs modulating brain NLRP3 inflammasome’s function.

(**A**) Activation.			
**RNAs**	**Model**	**Mechanisms**	**References**
LncRNA-Cox2	Murine microglia	↑ ^§^ Transcription of NLRP3 and ASC TLR-mediated signaling pathways Autophagy block Microglia activation	[180,181,188]
LncRNA-Meg3	Murine microglia	miR-7a-5 downregulation	[189]
miR-141	Brain tissue of diabetic mice	NF-ĸB-mediated NLRP3 expression	[190]
Exo-miR-124 Exo-miR-146a Exo-miR-155	LPS-primed N9 microglia cells	↑ TLR4/TLR2/NF-ĸB axis	[191]
miR-193	Murine brain cortex Murine microglia	↑ Expression of NLRP3, ASC, cleaved caspase-1 and mature IL-1β	[192]
miR-590-3	In silico AD patients’ data	Promoted neurons’ death via AMPK signaling	[193]
P3Alu-RNAs	Primary human retinal pigment cells	ERK1/2 and NLRP3 activation, neurons’ death	[184]
(**B**) Inhibition.			
**RNAs**	**Model**	**Mechanisms**	**References**
circRNA_003564	Spinal cord injury (rat model)	↓ § NLRP3, caspase-1, mature IL-1β, Il-18, GsdmD ↓ Pyroptosis	[187]
LncRNA-Meg3	Rat hippocampal neuronal model of temporal epilepsy	PI3K/AKT/mTOR pathway activation	[194]
miR-7	Murine neural stem cells	NLRP3/caspase-1 suppressor	[195,196]
Exo-miR-21	APP/PS1 2xTg AD-model mouse	Improved memory	[197]
miR-22, Exo-miR-22	APP/PS1 2xTg AD-model mouse PC12 cells	Downregulated NLRP3	[198,199]
Exo-miR-23b	Rat model of intracerebral hemorrhage	Antioxidant effects via PTEN/NRF2 inhibition	[200]
miR-29c-3p Exo-miR-29c-3p	PC12 cells AD-model rat	Suppression of BACE1, p-Tau, and pyroptosis via Wnt/β-catenin pathway	[201,202]
miR-152	Microglial BV2 cell Hippocampal neuronal HT22 cell line Rat model of intracerebral hemorrhage	TXNIP-mediated block of NLRP3 activation	[203]
Exo-miR-188-3p	PD-model mouse MN9D dopaminergic neuronal cells	Suppression of NLR3/pyroptosis	[204]
miR-194-5p	Rat model of intra- cerebral hemorrhage	Blocked NLRP3/TRAF6 interaction	[205]
miR-223-3p	Serum samples from PD, AD, and MCI patients, and healthy controls	Negative NLRP3 regulation	[206]
miR-374a-5p	Rat model of hypoxic-ischemia encephalopathy	Suppressor of SMAD6/NLRP3 in microglia	[207]

^§^ ↑ = increased; ↓ = decreased.

**Table 3:** Inhibitors of brain NLRP3 inflammasome.

Compound [References]	IUPAC Name	Main Molecular Activity	Main Biological Activity	Experimental Model
**17β-Estradiol (E2)** [223,224,225] **See also Box 1**	(8R,9S,13S,14S,17S)-13-methyl-6,7,8,9,11,12,14,15,16,17-decahydrocyclopenta[a]phenanthrene-3,17-diol	Ligand for estrogen receptor-*α* (ER-*α*) and -*β* (ER-*β*), and for G-protein coupled receptor 1 (GPER1)	↓^§^ NLRP3, ASC, cleaved caspase-1, IL-1β ↓§ M1 microglia ↑ M2 microglia	Male SOD1(G93A) ALS-model mice Global brain ischemia-model rodents
**A43879** [226] **See also Box 2**	3-[[5-(2,3-dichlorophenyl)-tetrazol-1-yl]methyl]pyridine hydrochloride	P2X_7_ purinergic receptor antagonist	↓ P2X_7_ receptor signaling ↓ NLRP3	Spinal cord injury-model animal
**Adiponectin** [227]	Protein	Ligand for Adipo-R1 and Adipo-R2 receptors	↓ NLRP3, IL-1β, IL-18 ↑ Autophagy via AMPK pathway	Intracerebral hemorrhage-model rat
**Amifostine** [228]	2-(3-aminopropylamino)ethyl-sulfanylphosphonic acid	Protects against the DNA-damaging effects of ionizing radiations and chemotherapy drug-induced ROS	↓ ROS, pyroptosis	Experimental autoimmune encephalomyelitis (EAE)-model rat
**α1-Antitrypsin (A1AT)** [128]	Protein	Protease inhibitor	↓ Aβ_1–42_-driven NLRP3 activation	Mouse primary cortical astrocytes
**Anfibatide** [229,230]	Dimeric protein	Antagonist of the glycoprotein Ib IX-V (GPIb) complex	↓ NLRP3/NF-κB axis, cleaved caspase-1 and -3, and Bax ↑ Bcl2	Cerebral HI/R injury-model rat
**Atorvastatin** [231]	(3*R*,5*R*)-7-[2-(4-fluorophenyl)-3-phenyl-4-(phenylcarbamoyl)-5-propan-2-ylpyrrol-1-yl]-3,5-dihydroxyheptanoic acid	Inhibitor of hydroxymethylglutaryl-coenzyme A (HMG-CoA) reductase	↓ NLRP3/NF-κB signaling axis	Surgery-induced BBB disruption in aged mice
**Bay117082** [232]	(E)-3-(4-methylphenyl)-sulfonylprop-2-enenitrile	Calcium channel blocker	↓ ATPase activity of NLRP3	Spinal cord injury-model animal
**BPBA** [233]	(2-[2-(benzo[d]thiazol-2-yl) phenyl-amino] benzoic acid)	Inhibitor of self- and Cu^2+^- or Zn^2+^-induced Aβs aggregation	↓ Aβs aggregation and neurotoxicity ↓ NLRP3 and IL-1β	Aβ-induced paralysis in transgenic *Caenorabditis elegans*
**Caffeine** [234]	1,3,7-trimethypurine-2,6-dione	Antagonist of all adenosine receptor subtypes (A1, A2a, A2b, A3) in the CNS PDE inhibitor	↓ Rapamycin (mTOR) axis and Bax ↑ Autophagy	EAE-model C57BL/6 mice Mouse microglia BV2 microglial cells
**Calcitriol** [235]	(1R,3S,5Z)-5-[(2E)-2-[(1R,3aS,7aR)-1-[(2R)-6-hydroxy-6-methylheptan-2-yl]-7a-methyl-2,3,3a,5,6,7-hexahydro-1H-inden-4-ylidene]ethylidene]-4-methylidenecyclohexane-1,3-diol	Ligand for vitamin D receptors	↓ ROS, NLRP3, caspase-1, IL-1β, CX3CR1, CCL17, Tbx21 ↓ Spinal cord demyelination	EAE-model C57BL/6 mice
**Choline** [236]	2-hydroxyethyl-(trimethyl)azanium	Methyl donor Ligand for choline transporters, CTL1 included	↓ NLRP3, Aβs deposition, and microgliosis	APP/PS1 AD-model mice
**Dapansutrile** **(i.e., OLT1177)** [237]	3-methylsulfonyl propanenitrile	Direct NLRP3 ATPase inhibitor	↓ Microglia activation and Aβs plaque numbers in the cerebral cortex ↓ IL-1β and IL-6 ↑ Dendritic spine density Successful Phase I clinical trial	APP/PS1 AD-model mice
**Dexmedetomidine (Dexm)** [96,238]	5-[(1S)-1-(2,3-dimethylphenyl)ethyl]-1H-imidazole	Specific and selective α-2 adrenoceptor agonist	↓ NF-κB and proinflammatory cytokines via miR-340 upregulation ↑ Autophagy	LPS-stimulated BV2 microglia cells
**Dihydromyricetin** [239]	(2*R*,3*R*)-3,5,7-trihydroxy-2-(3,4,5-trihydroxyphenyl)-2,3-dihydrochromen-4-one	Antioxidant, anti-binge hangover, and anti-cancer activity	↓ NLRP3 ↑ Aβ clearance ↑ Expression of neprilysin ↑ M2 microglial phenotype	APP/PS1 AD-model mice
**A-68930** [240]	1-(aminomethyl)-3-phenyl-3,4-dihydro-1*H*-isochromene-5,6-diol;hydrochloride	Potent and selective Dopamine D1-like receptor agonist	↓ NLRP3 activation	LPS-induced systemic inflammation mouse model
**Bromocriptine**	(6aR,9R)-5-bromo-N-[(1S,2S,4R,7S)-2-hydroxy-7-(2-methylpropyl)-5,8-dioxo-4-propan-2-yl-3-oxa-6,9-diazatricyclo[7.3.0.02,6]dodecan-4-yl]-7-methyl-6,6a,8,9-tetrahydro-4H-indolo[4,3-fg]quinoline-9-carboxamide	Dopamine D2 receptor agonist	↑ NLRP3 ubiquitination via cAMP	Neurotoxin MPTP-treated mice
**Dopamine** [226]	4-(2-aminoethyl)benzene- 1,2-diol	Agonist for the five Dopamine receptor subtypes (D1, D2, D3, D4, D5)	↓ IL-1β and IL-18 secretion	Spinal cord injury-model rat
**LY171555**	(4aR,8aR)-5-propyl-1,4,4a,6,7,8,8a,9-octahydropyrazolo[3,4-g]quinoline;hydrochloride	Specific dopamine D2 receptor agonist		
**Quinerolane**	(5aR,9aR)-6-propyl-5a,7,8,9,9a,10-hexahydro-5H-pyrido[2,3-g]quinazolin-2-amine	Dopamine D2 and D3 receptors agonist		
**EC144** [241]	5-[2-amino-4-chloro-7-[(4-methoxy-3,5-dimethylpyridin-2-yl)methyl]pyrrolo[2,3-d]pyrimidin-5-yl]-2-methylpent-4-yn-2-ol	Selective inhibitor of heat shock protein 90 (HSP90)	↓ IL-1β and IL-18	Peritonitis-model animal
**Echinacoside** [242]	[(2R,3R,4R,5R,6R)-6-[2-(3,4-dihydroxyphenyl)ethoxy]-5-hydroxy-2-[[(2R,3R,4S,5S,6R)-3,4,5-trihydroxy-6-(hydroxymethyl)oxan-2-yl]oxymethyl]-4-[(2S,3R,4R,5R,6S)-3,4,5-trihydroxy-6-methyloxan-2-yl]oxyoxan-3-yl] (E)-3-(3,4-dihydroxyphenyl)prop-2-enoate	Neuroprotective effects via undefined upstream mechanisms	↓ NLRP3, NF-κB-p65, and ROS	Spinal cord injury-model animal LPS-treated BV2 microglial cells
**Ellagic acid** [243]	6,7,13,14-tetrahydroxy-2,9-dioxatetracyclo[6.6.2.04,16.011,15]hexadeca-1(15),4,6,8(16),11,13-hexaene-3,10-dione	ATP-competitive inhibitor of constitutively active CK2 Ser/Thr protein kinase	↓ caspase-1, IL-6, IL-10, IL-17A, TNF-α, GFAP, and Iba1	EAE-model mouse
**Fimasartan** [244]	2-[2-butyl-4-methyl-6-oxo-1-[[4-[2-(2H-tetrazol-5-yl) phenyl]phenyl]methyl]pyrimidin-5-yl]-N,N-dimethylethanethioamide	Angiotensin II receptor antagonist	↓ NLRP3/ASC/caspase-1 and NF-κB pathways	Intracerebral hemorrhage-model rat Hemolysate-treated BV2 microglia
**Fluoxetine** [245]	N-methyl-3-phenyl-3-[4-(trifluoromethyl)phenoxy]propan-1-amine	Serotonin reuptake inhibitor	↓ NF-κB, TLR-4, NLRP3, caspase-1, TNF-α, IL-1β ↓ AChE activity, Aβ, Tau protein, MDA	Depression- and AD-model animals
**Ghrelin** [246]	(4S)-4-[[(2S)-1-[(2S)-2-[[(2S)-2-[[(2S)-2-[[(2S)-2-[[(2S)-2-[(2-aminoacetyl)amino]-3-hydroxypropanoyl]amino]-3-hydroxypropanoyl]amino]-3-phenylpropanoyl]amino]-4-methylpentanoyl]amino]-1-oxopropan-2-…………………………………. yl]amino]-5-oxopentanoic acid	Ligand for GHS-R1a receptor	↓ NF-κB/NLRP3 axis, IL-6, COX2, TNF-α, NOS-2, and pyroptosis	EAE-model animal
**Glibenclamide** [247,248]	5-chloro-N-[2-[4-(cyclohexylcarbamoylsulfamoyl)phenyl]ethyl]-2-methoxybenzamide	Classic K_ATP_ channel blocker	ATP-sensitive K^+^ channel inhibitor ↓ NLRP3 ↓ Release of HSP70 ↓ NLRP3, GsdmD-cleavage, ↓ Oxidative stress, demyelination, axon degeneration	Morphine-induced neuroinflammation animal and cellular models Hexanendione-induced neurotoxicity-model animal
**HU-308** [249]	[(1R,4R,5R)-4-[2,6-dimethoxy-4-(2-methyloctan-2-yl)phenyl]-6,6-dimethyl-2-bicyclo[3.1.1]hept-2-enyl]methanol	Activator of cannabinoid receptor 2	↑ Autophagy	BV2 microglia cells EAE-model animals
**Indomethacin** [250]	2-[1-(4-chlorobenzoyl)-5-methoxy-2-methylindol-3-yl]acetic acid	Prostaglandin G/H synthase 2 or cyclo-oxygenase (COX) enzyme inhibitor	↓ NLRC4 and NLRP3 genes ↓ IL-1β, caspase-1, and p-Taues	Streptozotocin (STZ)-induced AD-like model
**Inzomelid** [251]	1-(1,2,3,5,6,7-hexahydro-s-indacen-4-yl)-3-(1-propan-2-ylpyrazol-3-yl)sulfonylurea	Nonspecific and reversible inhibitor of the cyclo-oxygenase (COX) enzyme or prostaglandin G/H synthase	↓ NLRP3	ClinicalTrial.gov NCT04015076
**JC124** [252]	5-chloro-2-methoxy-N-[2-[4-(methylsulfamoyl)phenyl]ethyl]benzamide)	Specific inhibitor of expression of NLRP3 and its adaptor protein ASC	↓ NLRP3, ASC, IL-1β, TNFα, NOS-2, caspase-1, and pyroptosis	Traumatic brain injury in male rats
**Ketamine** [253]	2-(2-chlorophenyl)-2-(methylamino)cyclohexan-1-one	NMDA receptors antagonist	↓ NF-κB, NLRP3, ASC, caspase-1, IL-1β ↑ Autophagy	Depressive-like-model rat
**KPT-8602** [254]	(E)-3-[3-[3,5-bis(trifluoromethyl)phenyl]-1,2,4-triazol-1-yl]-2-pyrimidin-5-ylprop-2-enamide	Exportin 1 (XPO1) nuclear transport inhibitor	↓ Exportin 1 ↓ NLRP3/NF-κB signaling axis	LPS-treated macrophages LPS-induced inflammation mouse model MPTP mouse model of PD
**Licochalcone B** [255]	(E)-3-(3,4-dihydroxy-2-methoxyphenyl)-1-(4-hydroxyphenyl)prop-2-en-1-one	Specific inhibitor of NEK7-NLRP3 interaction	↓ Canonical and non-canonical NLRP3 inflammasome activation	Murine macrophages Mouse models of LPS-induced septic shock, peritonitis, and non-alcoholic steatohepatitis
**Manoalide** [256,257,258,259]	(2R)-2-hydroxy-3-[(2R,6R)-6-hydroxy-5-[(E)-4-methyl-6-(2,6,6-trimethylcyclohexen-1-yl)hex-3-enyl]-3,6-dihydro-2H-pyran-2-yl]-2H-furan-5-one	Inhibitor of NEK7-NLRP3 activating interaction	↓ Canonical and non-canonical NLRP3 inflammasome activation	EAE-model animal
**MCC950** **(i.e., CRID3)** [222,260]	1,2,3,5,6,7-hexahydro-s-indacen-4-ylcarbamoyl-[4-(2-hydroxypropan-2-yl)furan-2-yl]sulfonylazanide	Selectively and specifically binds NLRP3 NATCH domain hindering Walker B motif function thereby inhibiting NLRP3 conformational modifications and oligomerization	↓ NLRP3 ↑ Aβ-phagocytic capability of microglia ↓ IL-1β, IL-18, TNF-α, NLRP3, ASC, cleaved caspase-1, Iba1-, and GFAP-positive cells ↑ BDNF and PSD95 expression	APP/PS1 transgenic AD-model mouse LPS + ATP-induced microglia Perioperative neurocognitive disorders-model mice
**Mefenamic,** **Tolfenamic,** **Flufenamic,** **Meclofenamic acids** [261]	2-(2,3-dimethylanilino)benzoic acid 2-(3-chloro-2-methylanilino)benzoic acid 2-[3-(trifluoromethyl)anilino]benzoic acid 2-(2,6-dichloro-3-methylanilino)benzoic acid	Cyclooxygenase (COX) inhibitors Cl^-^ channel inhibitors	↓ NLRP3 and IL-1β processing and release	LPS-primed primary bone marrow-derived macrophages
**Melatonin** [262,263,264,265,266]	N-[2-(5-methoxy-1H-indol-3-yl)ethyl]acetamide	Natural hormone of the pineal gland acting through its receptors	↑ TFEB nuclear translocation ↑ mitophagy ↓ NLRP3, IL-18, IL-6, and IL-1β ↓ ROS ↑ Sirtuin 1 ↑ α7-nAChR-mediated “autophagic flux”	Aβ _25–35_-treated SH-SY5Y cells APP/PS1 AD-model mice Chronic Gulf War syndrome
**Metformin (MET)** [267]	3-(diaminomethylidene)-1,1-dimethylguanidine	AMP-activated protein kinase (AMPK) agonist	↓ NF-κB signaling pathway ↑ Sirtuin 1 ↓ NLRP3-mediated ECs pyroptosis	LPS-stimulated lung tissues and pulmonary endothelial cells
**Milrinone** [268]	6-methyl-2-oxo-5-pyridin-4-yl-1H-pyridine-3-carbonitrile	Inhibitor of phosphodiesterase III	↑ cAMP ↓ TLR4/MyD88/NF-κB axis ↓ IL-1β, IL-6, TNF-α ↓ Aβ, p-Tau, ROS	LPS/Aβ-treated BV2 microglial cells APP/PS1 AD-model mouse
**Minocycline** [269,270]	(4*S*,4*aS*,5*aR*,12*aR*)-4,7-bis(dimethylamino)-1,10,11,12*a*-tetrahydroxy-3,12-dioxo-4*a*,5,5*a*,6-tetrahydro-4*H*-tetracene-2-carboxamide	Caspase-1 negative modulator	↓ TLR-2, MyD88, NLRP3/NF-κB axis, IL-1β	AD-like dementia-model mouse
**Mitoquinone** **(MitoQ)** [271]	10-(4,5-dimethoxy-2-methyl-3,6-dioxocyclohexa-1,4-dien-1-yl)decyl-triphenylphosphanium	Selectively accumulates inside mitochondria with anti-oxidant action	↓ Mitochondrial ROS, NLRP3 activation, IL-1β, and IL-18 ↑ M2 phenotype microglia	Intracerebral hemorrhage-model mouse FeCl_2_-treated microglia
**N-acetylcysteine** [272]	(2R)-2-acetamido-3-sulfanylpropanoic acid	Stimulator of glutathione synthetase	↓ ROS ↑ NRF2-induced NAD(P)H quinone dehydrogenase 1 (NQO1)	Ischemic stroke-model rat
**Nafamostat** **mesylate** [273]	(6-carbamimidoyl naphthalen-2-yl) 4-(diaminomethyl-ideneamino)benzoate	Synthetic inhibitor of serine proteases with a wide spectrum of activity	↓ NLRP3/NF-κB signaling ↓ TNF-α, IL-1β, NOS-2, COX-2, IL-18	Stroke-model animal
**NT-0796** [274]	unknown	Orally available brain- penetrant NLRP3 inhibitor	↓ NLRP3	ANZCTR.org.au ACTRN126210010828-97
**Phenyl vinyl** **sulfone** [275]	ethenylsulfonylbenzene	Cysteine protease inhibitor	↓ NLRP3-mediated IL-1β release	LPS+ATP-treated J774A.1 cells LPS intraperitoneally injected C57BL/6 mouse
**Phoenixin-14** [276] **See also Box 1**	protein	Ligand for the multiple function G protein-coupled receptor GPR173	↓ HMGB1-mediated NLRP3 activation ↓ IL-1β and IL-18	LPS-treated mouse primary astrocytes
**Pramipexole** [277]	6S)-6-N-propyl-4,5,6,7-tetrahydro-1,3-benzothiazole-2,6-diamine	Dopamine-D3 receptors agonist	↑ Autophagy ↓ NLRP3, ASC, cleaved caspase-1 IL-1β, IL-18	LPS+ATP-stimulated primary mouse astrocytes PD-model mouse
**Prednisone (PDN)** [278]	(8S,9S,10R,13S,14S,17R)-17-hydroxy-17-(2-hydroxyacetyl)-10,13-dimethyl-6,7,8,9,12,14,15,16-octahydrocyclopenta[a]phenanthrene-3,11-dione	Glucocorticoid receptor agonist	↓ NLRP3 activation ↓ TNF-α, CCL8, CXCL10, CXCL16 ↓ astrocytes and microglia activation	Cuprizone (CPZ)- induced demyelination-model mouse
**Resolvin D1** [279] **See also Box 1**	(4Z,7S,8R,9E,11E,13Z,15E,17S,19Z)-7,8,17-trihydroxydocosa-4,9,11,13,15,19-hexaenoic acid	Ligand for *N*-formyl peptide receptor-2 and GPR-32	↑ A20 expression ↓ NLRP3/NF-κB axis	Subarachnoid hemorrhage-model rat
**Sildenafil** [280]	5-[2-ethoxy-5-(4-methylpiperazin-1-yl)sulfonylphenyl]-1-methyl-3-propyl-6H-pyrazolo[4,3-d]pyrimidin-7-one	3′,5′-cyclic GMP (cGMP)-specific phosphodiesterase inhibitor	↓ NLRP3 ↓ Hippocampal Aβ_1–40_ and Aβ_1–42_ ↑ Brain cGMP levels	APP/PS1 AD-model mouse
**TAK-242** **(CLI-095)** [103,281]	(R)-Ethyl 6-(N-(2-chloro-4-fluorophenyl)sulfamoyl)cyclohex-1-enecarboxylate	TLR-4 signal transduction inhibitors	↓ TLR-4-NF-κB-caspase-11 axis ↓ NLRP3, IL-1β, and IL-18	Methamphetamine-treated mouse and primary astrocytes Aβ_1–42_-treated BV2 microglia and HT-22 neurons
**1,2,4-TTB** [282]	1,2,4-Trimethoxybenzene	Inhibitor of NLRP3 oligomer formation	↓ Nigericin- or ATP-mediated NLRP3 activation	Murine bone marrow-derived macrophages (BMDMs) Primary mouse microglia EAE-model mice
**Urolithin A** [283]	3,8-dihydroxybenzo[c]chromen-6-one	Gut microflora processed derivative of ellagic acid	↓ NLRP3 activation via mitophagy promotion in microglia	LPS- or MPTP-treated BV2 microglial cells MPTP PD-model mouse
**VX-765** [284]	(2S)-1-[(2S)-2-[(4-amino-3-chlorobenzoyl)amino]-3,3-dimethylbutanoyl]-N-[(2R,3S)-2-ethoxy-5-oxooxolan-3-yl]pyrrolidine-2-carboxamide	Competitive inhibitor of ICE/caspase-1 (active metabolite: VRT-043198)	↓ NLRP3/caspase-1/GsdmD pathway	APP/PS1 AD-model mice BV2 microglial cells

^§^ ↑ = increased; ↓=decreased.

**Table 4:** Brain NLRP3 inflammasome downregulation by officinal plants agents/extracts.

(**A**) Agents.				
**Natural Compounds and Sources**	**Chemical Class**	**Biological Activities**	**Experimental Models**	**References**
**Andrographolide** from the roots and leaves of the plant Creat or Green chireta (*Andrographis paniculata* Wall. ex Nees)	labdane diterpenoid	↓§ P2X_7_ receptor signaling ↓ HMGB1-induced TLR-4-NFκB signaling	LPS-activated mixed glial cells LPS-treated mouse	[285] see also Box 2
**Artesunate/Artemisinin** from *Artemisiae Iwayomogii* Herba	sesquiterpene lactone	↓ Inflammatory response and neuron death ↑^§^ Expression of BDNF, GDNF, and NT-3 neurotrophins	Traumatic brain injury-model mouse LPS-stimulated BV-2 microglial cells LPS-treated mouse	[286,287]
**Astragaloside IV** from *Astragalus membranaceus* (i.e., Huangqi)	pentacyclic triterpenoid	Antioxidant activity	Transient cerebral ischemia/reperfusion (I/R)-model mice	[288]
**Baicalin** from the root of *Scutellaria baicalensis* Georgi	flavonoid	↓ TLR-4/NF-κB/NLRP3 axis	APP/PS1 AD-model mice LPS/Aβ-stimulated BV2 microglial cells	[289]
**Benzyl isothiocyanate** from cruciferous vegetables	benzene	↓ NLRP3 activation via mitochondria- generated ROS inhibition ↓ NF-κB signaling	LPS-induced BV2 microglial cells	[290]
**Bixin** from the seeds of the Achiote tree (i.e., *Bixa orellana*)	apocarotenoid	Suppression of thioredoxin-interacting protein (TXNIP)-NLRP3 activity	EAE-model mouse	[291]
**Carnosic acid (CA)** **Carnosol (CS)** from *Rosmarinus officinalis*	abietane-type tricyclic diterpenes	↑ KEAP1 (Kelch-like ECH-associated protein 1)/NRF2 (erythroid 2–related factor 2) transcriptional pathway activation ↓ HSP 90 inhibition	APP/PS1 AD-model mice Primary mouse bone marrow-derived macrophages	[292,293]
**Cucurbitacin B** from *Cucurbitaceae*	tetracyclic triterpene	↓ NLRP3, caspase-1 self-activation, and IL-1β release	Ischemia/reperfusion injury-model rat	[294]
**Dehydroisohispanolone** **diterpene (DT1)** from *Ballota hispanica* (Labiatae)	labdane (bicyclic diterpene)	↓ NF-κB and NLRP3 signaling	Nigericin-activated murine bone marrow-derived macrophages	[295]
**Demethylene-tetrahydroberberine (DMTHB)** from *Berberis vulgaris*, *Berberis* *aristata*	alkaloid	↓ NLRP3 inflammasome’s activation ↓ IL-6 signaling	AD-model mice	[296]
**Esculentoside A** from the roots of Indian pokeweed (i.e., *Phytolacca esculenta* Van Houtte)	triterpene saponin	↓ NF-κB, MAPKs and NLRP3 pathways	LPS-activated murine primary microglia cells and BV2 microglia cells	[297]
**Gastrodin** from rhizome of *Gastrodia elata* Blume	phenolic glycoside	↓ TLR4-NF-κB-NLRP3 axis and microglia-mediated neuroinflammation	LPS-treated rats	[298]
**Ginkgolide B** **(BN-52021)** from *Ginkgo biloba* and *Machilus wangchiana*	diterpenoid esters	↓ NLRP3 and microglia-mediated neuroinflammation ↑ NLRP3 autophagic degradation	Aβ_1–42_-induced BV2 cells LPS-primed BV2 cells senescence-accelerated male mouse prone 8 (SAMP8)	[299,300]
**Ginsenosides** **(Rb1, Rg1, Rg3, Rg5, Rh1, Compound K, Chikusetsusaponin IVa, Gintonin,** and **20(S)-Protopanaxatriol**) from *Panax ginseng* C.A. Meyer; *Panax quinquefolius* L. (i.e., American Ginseng); and *Panax japonicus* T. Nees	saponins	↓ NLRP3, NLRP1, AIM-2, and caspase-1 self-activation ↓ brain load of Aβs ↑ soluble (s)APP-α	AD in rodent models Depression-like behavior in rat model Post-traumatic stress disorder-like behavior in rodent model Stroke model High fat diet-model mouse	[301,302,303,304,305]
**Isoformononetin** from *Cicer arietinum* L. (chickpea)	methoxyisoflavone	↓ NLRP3, NLRP2, ASC, NFκB-p65, IL-1β, caspase-1 proteins, and ROS	Streptozotocin-treated rat	[306]
**Isoliquiritigenin** from the Chinese herbal medicine Glycyrrhiza (Guo Lao)	isoflavone	↓ NLRP3 ↑ NRF2-induced antioxidant activity	Hippocampal organotypic slice cultures after oxygen/glucose deprivation (OGD)	[307]
**Isosibiricin** from orange jasmine (i.e., *Murraya exotica* or *paniculata*)	coumarin	NLRP3-inhibition mediated by Dopamine D1/2 receptors	LPS-primed mouse BV-2 microglial cells	[308]
**Kaempferol** from several herbs in TCM	polyphenol flavonoid	↑ NLRP3 autophagic degradation	PD-model mouse LPS-primed BV-2 microglial cells	[309,310,311]
**β-Lapachone** from the Lapacho tree or Jacaranda (i.e., *Tabebuia Avellaneda* Lorentz)	benzochromenone	Antioxidant activity	Multiple sclerosis and AD-model animals	[312]
**Lychee seed polyphenols (LSPs)** from the *Litchi chinensis* tree	polyphenols	↑ Autophagy via the AMPK/mTOR/ULK1 axis ↑ Tight junctions’ expression ↑ LRP1 (i.e., low-density lipoprotein receptor-related protein 1), Beclin 1, and LC-3II proteins	Aβ-induced BV2 microglia cells APP/PS1 AD-model mouse	[313,314]
**Mangiferin** from the rhizome of *Anemarrhena asphodeloides* Bunge	C-glucoside xanthone	↓ NF-κB and NLRP3 signaling ↓ Microglial M1 polarization	LPS-induced BV2 cells	[315]
**Myricitrin** from the root bark of the tallow shrub (i.e., *Myrica cerifera* L.)	polyphenol hydroxy flavonoid	↓ NLRP3/Bax/Bcl2 axis NF-κB inactivation Antioxidant activity	Rat model of sepsis-linked encephalopathy Brain HI-model rat	[316,317]
**Neferine** from the green seed embryos of the lotus plant ( i.e., *Nelumbo nucifera* Gaertn)	bisbenzylisoquinoline alkaloid	↓ NLRP3-mediated neuronal pyroptosis	Neonatal HI brain damage model rat PC12 cells	[35]
**Nobiletin** from *Citrus* L. fruits	polymethoxylated flavonoid	↓ NLRP3 ↑ Autophagy via AMPK/mTOR/ULK1 axis	LPS-treated rat brain and BV2 cells	[318]
**Oleocanthal** from extra-virgin olive oil	phenylethanoid	↓ NLRP3 ↑ Autophagy via AMPK/mTOR/ULK1 axis	AD-model TgSwDI Mouse	[319]
**Oridonin** from *Isodon Rubescens* (Hemsl.) H. Hara	(1S,2S,5S,8R,9S,10S,11R,15S,18R)-9,10,15,18-tetrahydroxy-12,12-dimethyl-6-methylidene-17-oxapentacyclo[7.6.2.15,8.01,11.02,8]octadecan-7-one	Binds NLRP3’s NACHT domain blocking NEK-7-NLRP3 activating interaction ↓ NF-κB pathway, Aβ_1–42_-elicited neuroinflammation, and pyroptosis	Aβ_1–42_-induced AD mice	[320]
**Osthole** from the roots of various medicinal plants, including *Cnidium monnieri* L. and *Angelica pubescens* (Japan’s Shishiudo).	7-methoxy-8-(3-methylpent-2-enyl) coumarin	↓ NLRP3 ↓ brain load of Aβs	Rat model of chronic cerebral ischemic hypoperfusion	[321]
**Purpurin** from *Rubia tinctorum* L. **Rhein** from *Rheum rhabarbarum*	anthraquinones	↓ NLRP3, caspase-1 self-activation, and IL-1β release	AD-model animals Perirhinal cortex high-fat-diet-induced animal model	[322]
**Quercetin** (plant pigment)	flavonoid	Antioxidant activity ↓ NLRP3-pyroptosis-mediated IL-1β release ↑ Sirtuin	LPS-induced primary microglial cells and BV2 cells LPS-induced PD model mouse Depression-model mouse SAMP8 mice	[323,324]
**Sinomenine** from the roots of the climbing plant *Sinomenium acutum* (Thumb.)	alkaloid	Antioxidant and anti-inflammatory activity	EAE-model mouse	[325]
**Thonningianin A** from *Penthorum chinense*	ellagitannin polyphenol	↑ NLRP3 autophagic degradation via AMPK/ULK1 and Raf/MEK/ERK axis	In vitro and in vivo AD models, including, *C. elegans*, APP/PS1 mice, BV-2 cells, and PC-12 cells	[119]
**Withaferin** from Indian ginseng (i.e., *Withania somnifera*)	steroidal lactone	↓ Gene expression of NF-κB and associated neuroinflammatory molecules	SH-SY5Y cells transfected with APP plasmid (SH-APP)	[326]
(**B**) Herbal Extracts.				
**Herbal/Fruit Extract**	**Source**	**Biological Activity**	**Experimental Model**	**References**
**Açaí extract**	Berries of the *Euterpe oleracea* Mart. palm tree	Antioxidant activity	LPS- or nigericin-activated microglia (EOC 13.31) cells	[327]
***Crysanthemum indicum*** **extract (CIE)**	TCM (main components: chlorogenic acid, luteoloside, and 3,5-dicaffeoylquinic acid)	Antioxidant activity ↑ TrkB/Akt/CREB/BDNF and Akt/Nrf-2/ARE axes	H_2_O_2_-induced oxidative toxicity in hippocampal HT22 neuronal cell line	[328,329]
**Glycyrrhiza** **(Guo Lao)**	TCM (main components: licochalcone, isochalcone A, echinatin, isoliquiritigenin, and glycyrrhizin)	↓ NLRP3, TNF-α, IL-1β, and IL-18 ↑ AMPK/NRF2/antioxidant response element (ARE) signaling	LPS-induced chondrocyte pyroptosis LPS-induced macrophage cells Ischemic brain damage-model animal	[307,330]
**Kutki**	Ayurvedic medicine from rhizomes and roots of *Picrorhiza kurroa*	↓ NLRP3 and BACE-1 expression	5xFAD-model mice	[331]
**Pien-Tze-Huang**	TCM, including *Radix et Rhizoma Notoginseng*, *Moschus*, *Calculus Bovis*, and *Snake Gall*	↓ NLRP3 ↑ Autophagy via AMPK/mTOR/ULK1 axis	LPS-induced BV2 microglial cells cerebral ischemia/reperfusion impaired rats	[332]
**Tojapride**	TCM (main components: *Cyperus rotundus* L. (i.e., *Nagar motha* in India), *Perilla frutescens* L. (i.e., Basionym), and *Aurantii Fructus Immaturus* L., the natural flavanone glycosides *Naringin* and *Neohesperidin.*	↓ CaSR-mediated NLRP3 inflammasome’s activation	Esophageal epithelial cells (reflux esophagitis)	[333] see also Box 1
**Xingxiong**	Extract from *Ginkgo biloba* L. or *Ginkgo folium* L. and tetramethylpyrazine sodium chloride	↓ NLRP3 ↑Akt/NRF2 axis	Focal cerebral I/R damage	[334]
**Ze Lan**	Rhizomes or rootstalks of *Lycopus lucidus*	↓ NLRP3	H_2_O_2_-induced oxidative injury in rat embryo cortical neurons	[335]

^§^ ↑ = increased; ↓ = decreased.

## Data Availability

Not applicable.
